# Optimization algorithms as training approach with hybrid deep learning methods to develop an ultraviolet index forecasting model

**DOI:** 10.1007/s00477-022-02177-3

**Published:** 2022-02-24

**Authors:** A. A. Masrur Ahmed, Mohammad Hafez Ahmed, Sanjoy Kanti Saha, Oli Ahmed, Ambica Sutradhar

**Affiliations:** 1 School of Mathematics Physics and Computing, University of Southern Queensland, Springfield, QLD 4300 Australia; 2 Present Address: Department of Civil and Environmental Engineering, West Virginia University, PO BOX 6103, Morgantown, WV 26506-6103 USA; 3 Department of Civil and Environmental Engineering, Norwegian University of Science and Technology, Trondheim, Norway; 4 School of Modern Sciences, Leading University, Sylhet, 3112 Bangladesh

**Keywords:** Deep learning, Hybrid model, Solar ultraviolet index, Optimization algorithms, Public health

## Abstract

The solar ultraviolet index (UVI) is a key public health indicator to mitigate the ultraviolet-exposure related diseases. This study aimed to develop and compare the performances of different hybridised deep learning approaches with a convolutional neural network and long short-term memory referred to as CLSTM to forecast the daily UVI of Perth station, Western Australia. A complete ensemble empirical mode decomposition with adaptive noise (CEEMDAN) is incorporated coupled with four feature selection algorithms (i.e., genetic algorithm (GA), ant colony optimization (ACO), particle swarm optimization (PSO), and differential evolution (DEV)) to understand the diverse combinations of the predictor variables acquired from three distinct datasets (i.e., satellite data, ground-based SILO data, and synoptic mode climate indices). The CEEMDAN-CLSTM model coupled with GA appeared to be an accurate forecasting system in capturing the UVI. Compared to the counterpart benchmark models, the results demonstrated the excellent forecasting capability (i.e., low error and high efficiency) of the recommended hybrid CEEMDAN-CLSTM model in apprehending the complex and non-linear relationships between predictor variables and the daily UVI. The study inference can considerably enhance real-time exposure advice for the public and help mitigate the potential for solar UV-exposure-related diseases such as melanoma.

## Introduction

Solar ultraviolet (UV) radiation is an essential component in the sustenance of life on Earth (Norval et al. 2007). The UV irradiance consists of a small fraction (e.g., 5–7%) of the total radiation and produces numerous beneficial effects on human health. It has been in use since ancient times for improving body’s immune systems, such as strengthening bones and muscles (Juzeniene and Moan 2012) as well as treating various hard-to-treat skin diseases such as atopic dermatitis, psoriasis, phototherapy of localised scleroderma (Furuhashi et al. 2020; Kroft et al. 2008), and vitiligo (Roshan et al. 2020). UV-stimulated tanning has a positive mood changing and relaxing effect on many (Sivamani et al. 2009). Further, UV-induced nitrogen oxide (NO) plays a vital role in reducing human blood pressure (Juzeniene and Moan 2012; Opländer Christian et al. 2009).

UV light has also been widely used as an effective disinfectant in the food and water industries to inactivate disease-producing microorganisms (Gray 2014). Because of its effectiveness against protozoa contamination, the use of UV light as a drinking water disinfectant has achieved increased acceptance (Timmermann et al. 2015). To date, most of the UV-installed public water supplies are in Europe. In the United States (US), its application is mainly limited to groundwater treatment (Chen et al. 2006). However, its use is expected to increase in the future for the disinfection of different wastewater systems. Developing countries worldwide find it useful as it offers a simple, low-cost, and effective disinfection technique in water treatment compared to the traditional chlorination method (Mäusezahl et al. 2009; Pooi and Ng 2018).

The application of UV light has also shown potency in fighting airborne-mediated diseases for a long time (Hollaender et al. 1944; Wells and Fair 1935). For instance, a recent study demonstrated that a small dose (i.e., 2 mJ/cm^2^ of 222-nm) of UV-C light could efficiently inactivate aerosolized H1N1 influenza viruses (Welch et al. 2018). The far UV-C light can also be used to sterilize surgical equipment. Recently, the use of UV-C light as the surface disinfectant has been significantly increased to combat the global pandemic (COVID-19) caused by coronavirus SARS-CoV2. A recent study also highlighted the efficacy of UV light application in the disinfection of COVID-19 surface contamination (Heilingloh et al. 2020).

However, the research on UV radiation has also been a serious concern due to its dichotomous nature. UV irradiance can also have detrimental biological effects on human health, such as skin cancer and eye disease (Lucas et al. 2008; Turner et al. 2017). Chronic exposure to UV light has been reported as a significant risk factor responsible for melanoma and non-melanoma cancers (Saraiya et al. 2004; Sivamani et al. 2009) and is associated with 50–90% of these diseases. In a recent study, the highest global incidence rates of melanoma were observed in the Australasia region compared to other North American and European parts (Karimkhani et al. 2017). Therefore, it is crucial to provide correct information about the intensity of UV irradiance to the people at risk to protect their health. This information would also help people in different sectors (e.g., agriculture, medical sector, water management, etc.).

The World Health Organization (WHO) formulated the global UV index (UVI) as a numerical public health indicator to convey the associated risk when exposed to UV radiation (Fernández-Delgado et al. 2014; WHO 2002). However, UV irradiance estimation in practice requires ground-based physical models (Raksasat et al. 2021) and satellite-derived observing systems with advanced technical expertise (Kazantzidis et al. 2015). The installation of required equipment (i.e., spectroradiometers, radiometers, and sky images) is expensive (Deo et al. 2017) and difficult for remote regions, primarily mountainous areas. Furthermore, the solar irradiance is also highly impacted by many hydro-climatic factors, e.g., clouds and aerosol (Li et al. 2018; Staiger et al. 2008) and ozone (Baumgaertner et al. 2011; Tartaglione et al. 2020) that can insert considerable uncertainties into the available process-based and empirical models (details also given in the method section). Therefore, the analysis of sky images may also require extensive bias corrections, i.e., cloud modification (Krzyścin et al. 2015; Sudhibrabha et al. 2006), which creates further technical as well as computational burdens. An application of data-driven models can be helpful to minimize these formidable challenges. Specifically, the non-linearity into the data matrix can easily be handled using data-driven models that traditional process-based and semi-process-based models fail. Further, the data-driven models are easy to implement, do not demand high process-based cognitions (Qing and Niu 2018; Wang et al. 2018), and are computationally less burdensome.

As an alternative to conventional process-based and empirical models, applying different machine learning (ML) algorithms as data-driven models have proven tremendously successful because of the powerful computational efficiency. With technological advancement, computational efficiency has been significantly increased, and researchers have developed many ML tools. Artificial neural networks (ANNs) are the most common and extensively employed in solar energy applications (Yadav and Chandel 2014). However, many studies, such as the multiple layer perceptron (MLP) neural networks (Alados et al. 2007; Alfadda et al. 2018), support vector regression (SVR) (Fan et al. 2020; Kaba et al. 2017), decision tree (Jiménez-Pérez and Mora-López 2016), and random forest (Fouilloy et al. 2018) have also been extensively applied in estimating the UV erythemal irradiance. The multivariate adaptive regression splines (MARS) and M5 algorithms were applied in a separate study for forecasting solar radiation (Srivastava et al. 2019). Further, the deep learning network such as the convolutional neural network (CNN) (Szenicer et al. 2019) and the long short-term memory (LSTM) (Ahmed et al. 2021b, c; Huang et al. 2020; Qing and Niu 2018; Raksasat et al. 2021) are recent additions in this domain.

However, the UVI indicator is more explicit to common people than UV irradiance values. Further, only a few data-driven models have been applied for UVI forecasting. For example, an ANN was used in modeling UVI on a global scale (Latosińska et al. 2015). An extreme learning method (ELM) was applied in forecasting UVI in the Australian context (Deo et al. 2017). There have not been many studies that used ML methods to forecast UVI. Albeit the successful predictions of these standalone ML algorithms, they have architectural flaws and predominantly suffer from overfitting efficiency (Ahmed and Lin 2021). Therefore, the hybrid deep learning models receive increased interest and are extremely useful in predictions with higher efficiency than the standalone machine learning models. Hybrid models such as particle swarm optimization (PSO)-ANN, wavelet-ANN (Zhang et al. 2019), genetic algorithm (GA)-ANN (Antanasijević et al. 2014), Boruta random forest (BRF)-LSTM (Ahmed et al. 2021a, b, c, d), ensemble empirical mode decomposition (EEMD) (Liu et al. 2015), adaptive neuro-fuzzy inference system (ANFIS)-ant colony optimization (ACO) (Pruthi and Bhardwaj 2021) and (ACO)-CNN-GRU have been applied across disciplines and attained substantial tractions. However, a CNN-LSTM (i.e., CLSTM) hybrid model can efficiently extract inherent features from the data matrix than other machine learning models and has successfully predicted time series air quality and meteorological data (Pak et al. 2018). This study incorporates four feature selection algorithms (i.c., GA, PSO, ACO, and DEV) to optimize the training procedure and try different predictor variables selected by the feature selection algorithms. Adapting different feature selection approaches would give a diverse understanding of the predictors and effectively quantify the features of UVI. Moreover, integration of convolutional neural network as a feature extraction method gives a further improvement of UVI forecasting, as confirmed by numerous researchers (c 2021a; Ghimire et al. 2019; Huang and Kuo 2018; Wu et al. 2021). The application of such a hybrid model for predicting sequence data, i.e., the UVI for consecutive days, can be an effective tool with excellent predictive power. However, the forecasting of UVI with a CLSTM hybrid machine learning model is yet to be explored and was a key motivation for conducting this present study.

In this study, we employed a new model of EMD called complete ensemble empirical mode decomposition with adaptive noise (CEEMDAN) (Ahmed et al. 2021b; Prasad et al. 2018). In CEEMDAN-based decomposition, Gaussian white noise with a unit variance is added consecutively at each stage to reduce the forecasting procedure’s complexity (Di et al. 2014). Over the last few years, CEEMDAN techniques have been successfully implemented in forecasting soil moisture (Ahmed et al. 2021b; Prasad et al. 2018, 2019a, b), draught (Liu and Wang 2021), precipitation (Wang et al. 2022), and wind energy (Liang et al. 2020; Zhang et al. 2017). However, a previous version (i.e., EEMD) was used in forecasting streamflow (Seo and Kim 2016) and rainfall (Beltrán-Castro et al. 2013; Jiao et al. 2016; Ouyang et al. 2016). The machine learning algorithm used in the study is CLSTM, which has not been coupled with the EEMD or CEEMDAN to produce a UVI forecast system.

This study aims to apply a CLSTM hybrid machine learning model, which can exploit the benefits of both convolutional layers (i.e., feature extraction) and LSTM layers (i.e., storing sequence data for an extended period) and evaluate its ability to forecast the UVI for the next day efficiently. The model was constructed and fed with hydro-climatic data associated with UV irradiance in the Australian context. The model was optimized using ant colony optimization, genetic algorithms, particle swarm optimization, and differential evolutional algorithms. The model accuracy (i.e., efficiency and errors involved in UVI estimations) was assessed with the conventional standalone data-driven models’ (e.g., SVR, decision tree, MLP, CNN, LSTM, gated recurrent unit (GRU), etc.) performance statistics. The inference obtained from the modeling results was also tremendously valuable for building expert judgment to protect public health in the Australian region and beyond.

## Materials and methods

### Study area and UVI data

**Fig. 1:**
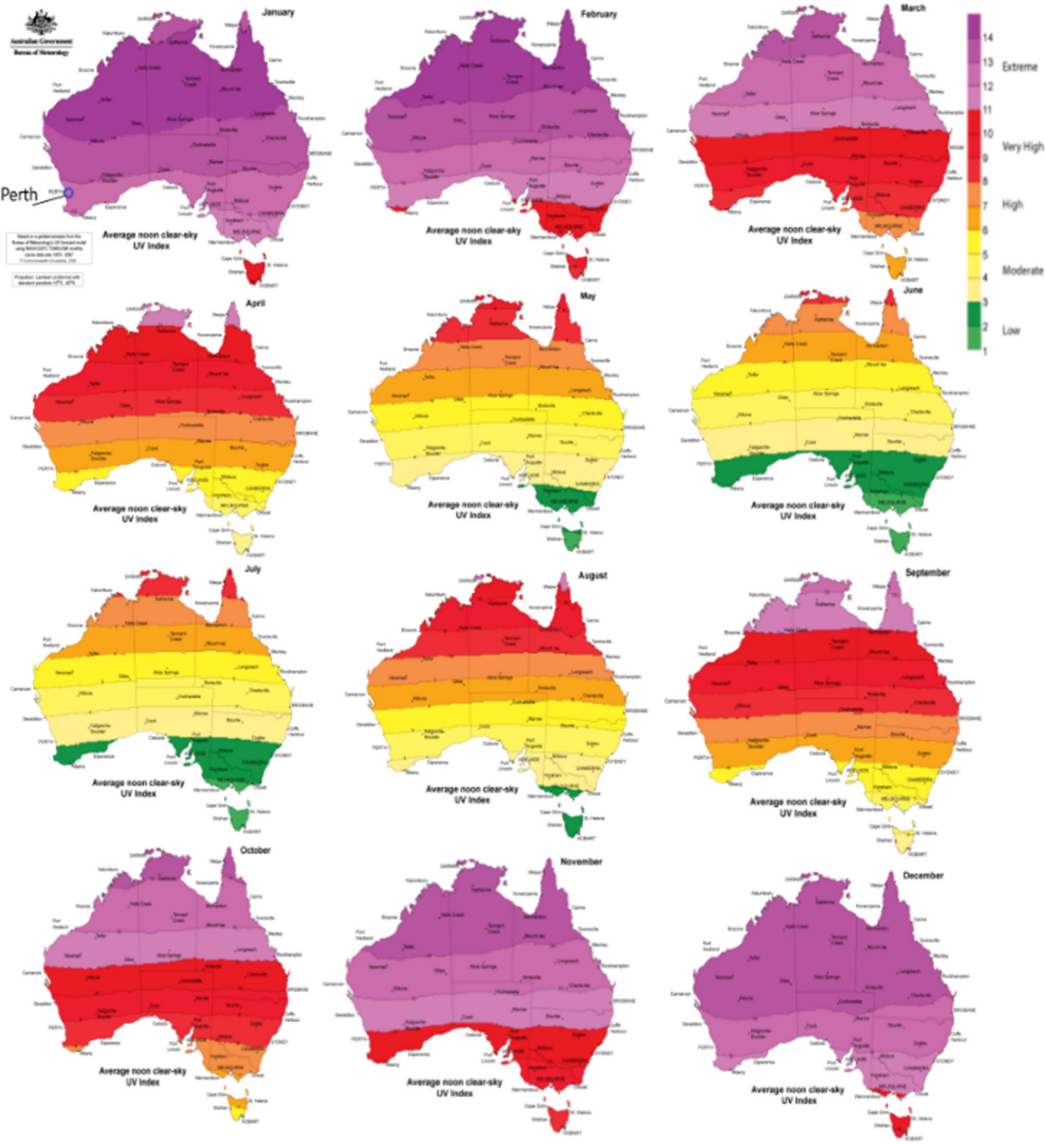
Study site (Perth, Australia) of the work and monthly average noon clear-sky UV index based on gridded analysis from the Bureau of Meteorology’s UV forecast model using NASA/GFSC TOMS OMI monthly ozone data sets between 1979 and 2007

The study assessed the solar ultraviolet index of Perth (Latitude: − 31.93° E and Longitude: 115.10° S), Western Australia. The Australian Radiation Protection and Nuclear Safety Agency (ARPANSA) provided the UVI data for Australia from https://www.arpansa.gov.au (ARPANSA 2021). Figure 1 shows the monthly UVI data, the location of Perth, and the assessed station. The figure shows that Perth has had low to extreme UV concentrations between 1979 and 2007. The summer season (December to February) had the most extreme UV concentration. In contrast, autumn (March to May) has a moderate to high UVI value, and winter (June to August) demonstrates a lower to moderate, and spring (September to November) has a higher to extreme UVI value in Perth.

**Fig. 2:**
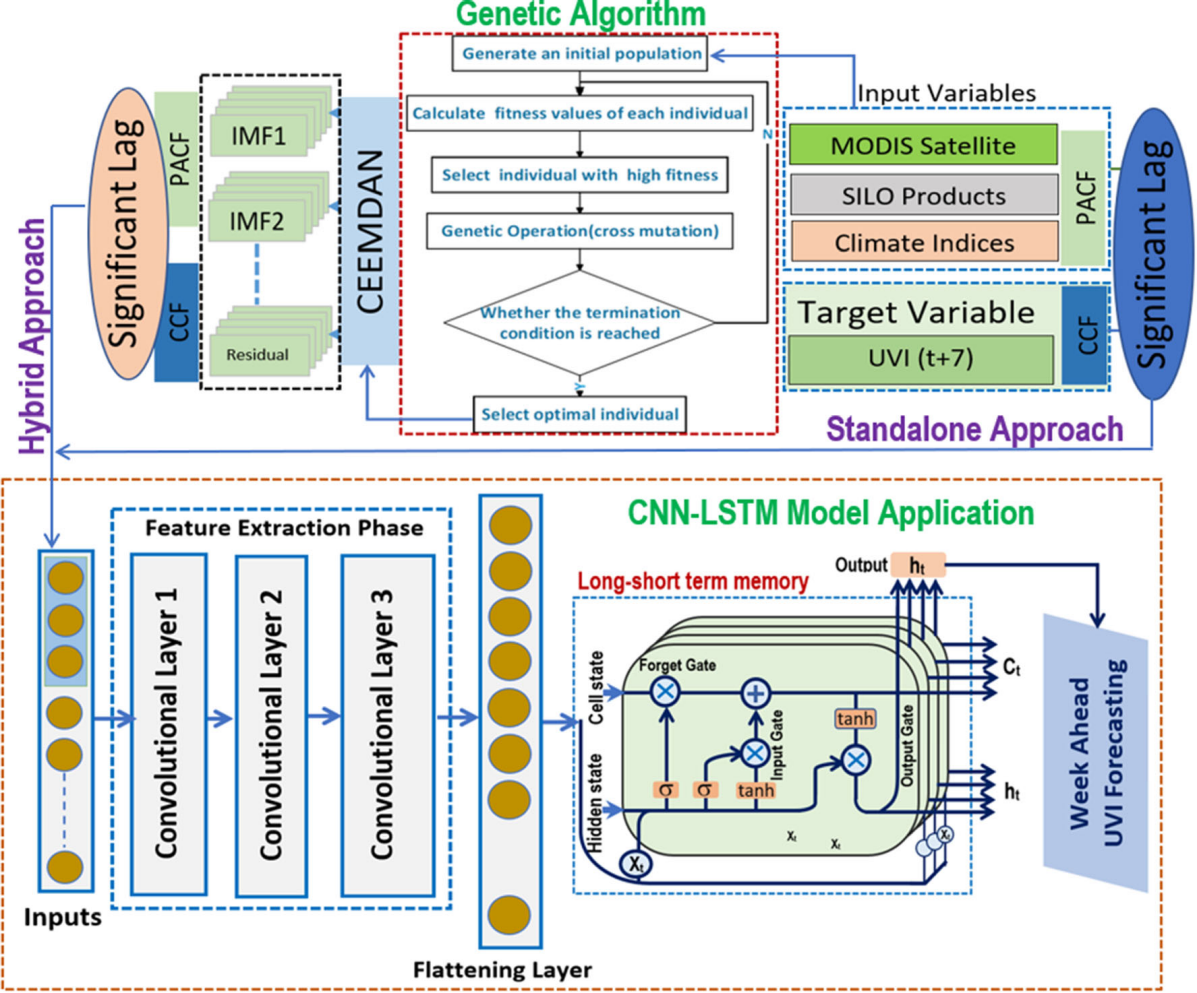
The developed model architecture of (Convolutional Neural Network, CNN) with the 4 layered long short term memory for a hybrid CNN-LSTM model to forecast a week daily maximum UV Index with Genetic Algorithm

Malignant melanoma rates in Western Australia are second only to those in Queensland, Australia’s most populated state (Slevin et al. 2000). Australia has the highest incidence of NMSC (Non-melanoma skin cancer) globally (Anderiesz et al. 2006; Staples et al. 1998). Approximately three-quarters of the cancer cases have basal cell carcinoma (BCC) and squamous cell carcinoma (SCC) types. These are attributed to the fair-skinned population’s high exposure to ambient solar radiation (Boniol 2016; McCarthy 2004). As a result, Australia is seen as a world leader in public health initiatives to prevent and detect skin cancer. Programs that have brought awareness of prevention strategies and skin cancer diagnoses have data to show that people act on their knowledge (Stanton et al. 2004). Several studies have found that decreasing sun protection measures are associated with a reduction in the rates of BCC and SCC in younger populations. They might have received cancer prevention messages as children (Staples et al. 2006). Considering the diversified concentration of UVI concentration, this study considers Perth an ideal study area (Fig. 2).

### Datasets of predictor variables

Three distinct data sources were used to collect the predictor variables in this analysis. The Moderate Resolution Imaging Spectroradiometer (MODIS) satellite datasets capture land surface status and flow parameters at regular temporal resolutions. These are supplemented by ground-based Scientific Information for Landowners (SILO) repository meteorological data for biophysical modeling and climate mode indices to help achieve Sea Surface Temperature (SST) over Australia. Geospatial Online Interactive Visualization and Analysis Infrastructure (GIOVANNI) is a geoscience data repository that provides a robust online visualization and analysis platform for geoscience datasets. It collects data from over 2000 satellite variables (Chen et al. 2010). The MODIS- aqua yielded 8 predictor variables for our study: a high-temporal terrestrial modeling system consisting of a surface state and providing daily products with a high resolution (250 m at nadir). A list of predictors of the MODIS Satellite can be obtained from the National Aeronautics and Space Administration (NASA) database (Giovanni 2021).

The surface UVI is influenced by atmospheric attenuation of incident solar radiation (Deo et al. 2017). The angle subtended from the zenith (θs) to the solar disc is another factor that affects the intensity of solar UV radiation (Allaart et al. 2004). The ultraviolet attenuation of clear-sky solar radiation is dependent on ozone and atmospheric aerosol concentrations, along with cloud cover (Deo et al. 2017). This implies that the measurements of biologically effective UV wavelengths are affected by total ozone column concentration. Incident radiation at the Earth’s surface is reduced by aerosols such as dust, smoke, and vehicle exhausts (Downs et al. 2016; Román et al. 2013). Moreover, Lee et al. (2009) found a significant correlation between UV solar radiation and geopotential height. Considering the direct influence of the predictors over ultraviolet radiation and UV index, this study collected ozone total column, aerosol optical depth (550 nm and 342.5 nm), geopotential height, cloud fraction, and combined cloud optical thickness data from the Geospatial Online Interactive Visualization and Analysis Infrastructure (GIOVANNI) repository.

Therefore, meteorological predictor variables (i.e., temperature, u- and v-winds) were significant while modeling UVI (Lee et al. 2009). Moreover, the cloud amount and diurnal temperature range have a strong positive correlation, while rainfall and cloud amount show a strong negative correlation (Jovanovic et al. 2011). Although overall cloud patterns agree with rainfall patterns across Australia, the higher-quality cloud network is too coarse to represent topographic influences accurately. Changes in the amount of cloud cover caused by climate change can result in long-term changes in maximum and minimum temperature. Owing to the relations of hydro-meteorological variables with UVI and their interconnections with cloud cover, the study selected nine meteorological variables from the Scientific Knowledge for Land-Owners (SILO) database to expand the pool of predictor variables, allowing for more practical application and model efficiency. SILO data are managed by Queensland’s Department of Environment and Research and can be obtained from https://www.longpaddock.qld.gov.au/silo.

Aerosol-rainfall relationships are also likely to be artifacts of cloud and cloud-clearing procedures. During the Madden–Julian Oscillation (MJO) wet phase, the high cloud’s value increases, the cloud tops rise, and increased precipitation enhances wet deposition, which reduces aerosol mass loading in the troposphere (Tian et al. 2008). The MJO (Lau and Waliser 2011; Madden and Julian 1971, 1994) dominates the intra-seasonal variability in the tropical atmosphere. A relatively slow-moving, large-scale oscillation in the deep tropical convection and baroclinic winds exists in the warmer tropical waters in the Indian and western Pacific Oceans (Hendon and Salby 1994; Kiladis et al. 2001; Tian et al. 2008). The study used the Real-time Multivariate MJO series 1 (RMM1) and 2 (RMM2) obtained from the Bureau of Meteorology, Australia (BOM 2020). Though RMM1 and RMM2 indicate an evolution of the MJO independent of season, the coherent off-equatorial behavior is strongly seasonal (Wheeler and Hendon 2004). Pavlakis et al. (2007, 2008) studied the spatial and temporal variation of long surface and short wave radiation. A high correlation was found between the longwave radiation anomaly and the Niño3.4 index time series over the Niño3.4 region located in the central Pacific.

Moreover, Pinker et al. (2017) investigated the effect of El Niño and La Nina cycles on surface radiative fluxes and the correlations between their anomalies and a variety of El Niño indices. The maximum variance of anomalous incoming solar radiation is located just west of the dateline. It coincides with anomalous SST (Sea surface temperature) gradient in the traditional eastern Pacific El Niño Southern Oscillation (ENSO). However, we derive the Southern Oscillation Index highly correlated with solar irradiance and mean Northern Hemisphere temperature fluctuations reconstructions (Yan et al. 2011). In North America and the North Pacific, land and sea surface temperatures, precipitation, and storm tracks are determined mainly by atmospheric variability associated with the Pacific North American (PNA) pattern. The modern instrumental record indicates a recent trend towards a positive PNA phase, which has resulted in increased warming and snowpack loss in northwest North America (Liu et al. 2017). This study used fifteen climate mode indices to increase the diversity. Table 1 shows the list of predictor variables used in this study.

**Table 1:** Description of global pool of 24 predictor variables used to design and evaluate hybrid CEEMDAN-CLSTM predictive model for the daily maximum UV Index forecasting

*MODIS-satellite*		
OTC	Ozone total column	DU
GH	Geopotential height (daytime)	–
AO	Aerosol optical depth 550 nm	–
AOD2	Aerosol optical depth 342.5 nm	–
TCW	Total column water vapour (daytime)	kg/m^2^
CF	Cloud fraction (daytime)	–
CP	Cloud pressure (daytime)	hPa
CCO	Combined cloud optical thickness (mean)	–
*SILO (ground-based observations)*		
T.Max	Maximum temperature	°C
T.Min	Minimum temperature	°C
Rain	Rainfall	mm
Evap	Evaporation	mm
Radn	Radiation	MJ m^−2^
VP	Vapour pressure	hPa
RHmaxT	Relative humidity at Temperature T.Max	%
RHminT	Relative Humidity at Temperature T.Min	%
FAO56	Morton potential evapotranspiration overland	mm
*SYNOPTIC-SCALE (climate mode indices)*		
Nino3.0	Average SSTA over 150°–90° W and 5° N–5° S	NONE
Nino3.4	Average SSTA over 170° E–120° W and 5° N–5° S	
Nino4.0	Average SSTA over 160° E–150° W and 5° N–5° S	
Nino1 + 2	Average SSTA over 90° W–80° W and 0°–10° S	
AON	Arctic oscillation	
AAO	Antarctic oscillation	
EPO	East Pacific oscillation	
GBI	Greenland blocking index (GBI)	
WPO	Western Pacific Oscillation (WPO.)	
PNA	Pacific North American Index	
NAO	North Atlantic oscillation	
SAM	Southern annular mode index	
SOI	Southern oscillation Index, as per Troup (1965)	
RMM1	Real-time multivariate MJO indices 1	
RMM2	Real-time multivariate MJO indices 1	

### Standalone models

#### Multilayer perceptron (MLP)

The MLP is a simple feedforward neural network with three layers and is commonly used as a reference model for comparison in machine learning application research (Ahmed and Lin 2021). The three layers are the input layer, a hidden layer with n-nodes, and the output layer. The input data are fed into the input layer, transformed into the hidden layer via a non-linear activation function (i.e., a logistic function). The target output is estimated, Eq. (1).

1 $$y = f\left( {\sum w^{T} x + b} \right)$$

where *w* = the vector of weights, *x*_*i*_ = the vector of inputs, *b* = the bias term; *f* = the non-linear sigmoidal activation function, i.e., $f\left( z \right) = \frac{1}{{1 + e^{ - z} }}$.

The computed output is then compared with the measured output, and the corresponding loss, i.e., the mean squared error (MSE), is estimated. The model parameters (i.e., initial weights and bias) are updated using a backpropagation method until the minimum MSE is obtained. The model is trained for several iterations and tested for new data sets for prediction accuracy.

#### Support vector regression (SVR)

The SVR is constructed based on the statistical learning theory. In SVR, a kernel trick is applied that transfers the input features into the higher dimension to construct an optimal separating hyperplane as follows (Ji et al. 2017):

2 $$f\left( x \right) = w.\varphi \left( x \right) + b$$

where *w* is the weight vector, *b* is the bias, and $\varphi \left( x \right)$ indicates the high-dimensional feature space. The coefficients *w* and *b*, which define the location of the hyperplane, can be estimated by minimizing the following regularized risk function:

3 $${\text{Minimize}}:\;\;\frac{1}{2}||w^{2} || + C\mathop \sum \limits_{1}^{n} \left( {\varepsilon_{i} + \varepsilon_{i}^{*} } \right)$$

$${\text{Subject}}\;{\text{to}}\;\;y_{i} - w \cdot \varphi \left( x \right) - b \le \varepsilon + \varepsilon_{i} ;\;\;w \cdot \varphi \left( x \right) + b - y_{i} \le \varepsilon + \varepsilon_{i}^{*} ;\;\varepsilon_{i} \le 0;\;\varepsilon_{i}^{*} \le 0$$

where *C* is the regularization parameter, $\varepsilon_{i}$ and $\varepsilon_{i}^{*}$ are slack variables. Equation (7) can be solved in a dual form using the Lagrangian multipliers as follows:

4 $${\text{Maximize}}:\;\; - \frac{1}{2}\mathop \sum \limits_{i = 1}^{n} \mathop \sum \limits_{j = 1}^{n} \left( {a_{i} - a_{i}^{*} } \right)\left( {a_{j} - a_{j}^{*} } \right)K\left( {x_{i} ,x_{j} } \right) - \mathop \sum \limits_{i = 1}^{n} \left( {a_{i} - a_{i}^{*} } \right) + \mathop \sum \limits_{1}^{i = n} \left( {a_{i} - a_{i}^{*} } \right)y_{i}$$

$${\text{Subject}}\;{\text{to}}\;\;\mathop \sum \limits_{i = 1}^{n} \left( {a_{i} - a_{i}^{*} } \right) = 0; \;a_{i} ,a_{i}^{*} \in \left[ {0,C} \right]$$

where $K\left( {x_{i} ,x} \right)$ is the non-linear kernel function. In this present study, we used a radial basis function (RBF) as the kernel, which is represented as follows:

$$K\left( {x_{i} ,x} \right) = exp\left( {\frac{{ - \left| {\left| {x - x_{i} } \right|} \right|^{2} }}{{2\sigma^{2} }}} \right)$$

where $\sigma$ is the bandwidth of the RBF.

#### Decision tree (DT)

A decision tree is a predictive model used for classification and regression analysis (Jiménez-Pérez and Mora-López 2016). As our data is continuous, we used it for the regression predictions. It is a simple tree-like structure that uses the input observations (i.e., x_1_, x_2_, x_3_, …, x_n_) to predict the target output (i.e., Y). The tree contains many nodes, and at each node, a test to one of the inputs (e.g., x_1_) is applied, and the outcome is estimated. The left/right sub-branch of the decision tree is selected based on the estimated outcome. After a specific node, the prediction is made, and the corresponding node is termed the leaf node. The prediction averages out all the training points for the leaf node. The model is trained using all input variables and corresponding loss; the mean squared error (MSE) is calculated to determine the best split of the data. The maximum features are set as the total input features during the partition.

#### Convolutional neural network (CNN)

The CNN model was developed initially for document recognition (Lecun et al. 1998) and used for predictions. Aside from the input and output layer, the CNN architecture has three hidden layers: convolutional, pooling, and fully connected. The convolutional layers abstract the local information from the data matrix using a kernel. The primary advantage of this layer is the implementation of weight sharing and spatial correlation among neighbors (Guo et al. 2016). The pooling layers are the subsampling layers that reduce the size of the data matrix. A fully connected layer is similar to the traditional neural network added at the final pooling layer after completing an alternate stack of convolutional and pooling layers.

#### Long short-term memory (LSTM)

An LSTM network is a unique form of recurrent neural network that stores sequence data for an extended period (Hochreiter and Schmidhuber 1997). The LSTM structure has three gates: an input gate, an output gate, and a forget gate. The model regulates all these three gates and determines how much data must be stored and transferred to the next steps from previous time steps. The input gate controls the input data at the current time as follows:

5 $$a_{l}^{t} = \mathop \sum \limits_{i = 1}^{I} w_{il} x_{i}^{t} + \mathop \sum \limits_{h = 1}^{H} w_{hl} b_{h}^{t - 1} + \mathop \sum \limits_{c = 1}^{C} w_{cl} s_{c}^{t - 1} ;b_{l}^{t} = f\left( {a_{l}^{t} } \right)$$

where $x_{i}^{t}$ = the input received from the *i*th node at time *t*; $b_{h}^{t - 1}$ = the result of the *h*th node at time *t* − 1; $s_{c}^{t - 1}$ = the cell state (i.e., memory) of the *c*th node at time *t* − 1. The symbol ‘*w*’ represents the weight between nodes, and the *f* is the activation function. The output gate transfers the current value from Eq. (5) to the output node, Eq. (6). Then, at the final stage, the current value is stored as the cell state in the forget gate, Eq. (7).

6 $$a_{w}^{t} = \mathop \sum \limits_{i = 1}^{I} w_{iw} x_{i}^{t} + \mathop \sum \limits_{h = 1}^{H} w_{hw} b_{h}^{t - 1} + \mathop \sum \limits_{c = 1}^{C} w_{cw} s_{c}^{t - 1} ;b_{w}^{t} = f\left( {a_{w}^{t} } \right)$$

7 $$a_{\emptyset }^{t} = \mathop \sum \limits_{i = 1}^{I} w_{i\emptyset } x_{i}^{t} + \mathop \sum \limits_{h = 1}^{H} w_{h\emptyset } b_{h}^{t - 1} + \mathop \sum \limits_{c = 1}^{C} w_{c\emptyset } s_{c}^{t - 1} ;b_{\emptyset }^{t} = f\left( {a_{\emptyset }^{t} } \right)$$

#### Gated recurrent unit (GRU) network

The GRU network is an LSTM variant having only two gates, such as reset and update gates (Dey and Salem 2017). The implementation of this network can be represented by the following equations, Eqs. (14–17):

8 $$z = \sigma \left( {W_{z} x_{t} + U_{z} h_{t - 1} + b_{z} } \right)$$

9 $$r = \sigma \left( {W_{r} x_{t} + U_{r} h_{t - 1} + b_{r} } \right)$$

10 $$m = \emptyset \left( {W_{m} x_{t} + U_{m} \left( {h_{t - 1} .r} \right) + b_{m} } \right)$$

11 $$h_{t} = \left( {1 - z} \right)h_{t - 1} + z \cdot m$$

where $\sigma$ = the sigmoidal activation function; $x_{t}$ = the input value at time *t*; $h_{t - 1}$ = the output value at time *t*-1; and the $W_{z}$, $U_{z}$,$W_{r}$, $U_{r}$,$W_{m}$, $U_{m}$ are the weight matrices for each gate and cell state. The symbols *r* and *z* represent the reset and update gates, respectively. $\emptyset$ is the activation function, and the dot [.] represents the element-wise dot product.

### The proposed hybrid model

#### CLSTM (or CNN-LSTM) hybrid model

In this paper, a deep learning method using optimization techniques is constructed on top of a forecast model framework. This study demonstrates how the CNN-LSTM (CLSTM) model, comprised of four-layered CNN, can be effectively used for UVI forecasting. The CNN is employed to integrate extracted features to forecast the target variable (i.e., UVI) with minimal training and testing error. Likewise, the CNN-GRU (CGRU) hybrid model is prepared for the same purpose.

### Optimization techniques

#### Ant colony optimization

Ant colony optimization (ACO) algorithm model is the graphical representation of the real ants’ behavior. In general, ants live in colonies, and they forage for food as a whole by communicating with each other using a chemical substance, the pheromones (Mucherino et al. 2015). An isolated ant cannot move randomly; they always optimize their way towards the food deposit to their nests by interacting with previously laid pheromones marks on the way. The entire colony optimizes their routes with this communication process and establishes the shortest path to their nests from feeding sources (Silva et al. 2009). In ACO, the artificial ants find a solution by moving on the problem graph. They deposit synthetic hormone pheromones on the graph so that upcoming artificial ants can follow the pattern to build a better solution. The artificial ants calculate the model’s intrinsic mode functions (IMFs) anticipation by testing artificial pheromone values against the target data. The probability of finding the best IMFs increases for every ant because of changing pheromones value throughout the IMFs. The whole process is just like an ant’s finding the optimal option to reach the target. The probability $p_{fi} \left( d \right)$ of selecting the shortest distance between the target and the IMFs of the input variable can be mathematically expressed as follows (Prasad 2019a, b):

12 $$p_{fi} \left( d \right) = \frac{{\left( {d_{i} + \Delta_{fi} \left( d \right)} \right)^{2} }}{{\left( {d_{i} + \Delta_{fi} \left( d \right)} \right)^{2} + \left( {d_{i} + \Delta_{ft} \left( d \right)} \right)^{2} }}$$

where $f \in \left\{ {1,2} \right\}$ denotes decision point, $i$ and $t$ express as short and long distance to the target at an instant $d$ is the total amount of pheromone $\Delta_{ft} \left( d \right)$. The probability of the longest path can be determined where $p_{fi} \left( d \right) + p_{ft} \left( d \right) = 1$. The testing update on the two branches is described as follows:

13 $$\Delta_{fi} \left( d \right) = \Delta_{fi} \left( {d - 1} \right) + p_{fi} \left( {d - 1} \right)a_{f} \left( {d - 1} \right) + p_{ki} \left( {d - 1} \right)a_{k} \left( {d - 1} \right)$$

14 $$\Delta_{ft} \left( d \right) = \Delta_{ft} \left( {d - 1} \right) + p_{ft} \left( {d - 1} \right)a_{f} \left( {d - 1} \right) + p_{kt} \left( {d - r} \right)a_{k} \left( {d - r} \right)$$

where $f,k \in \left\{ {1,2} \right\}$ and the value of $r$ represent the remainder in the model. $a_{f} \left( d \right)$ denotes the number of ants in the node $f$ at a certain period $d$ is given by:

15 $$a_{f} \left( d \right) = p_{ki} \left( {d - 1} \right)a_{k} \left( {d - 1} \right) + p_{kt} \left( {d - r} \right)a_{k} \left( {d - r} \right)$$

The ACO algorithm is the most used simulation optimization algorithm where myriad artificial ants work in a simulated mathematical space to search for optimal solutions for a given problem. The ant colony algorithm is dominant in multi-objective optimization as it follows the natural distribution and self-evolved simple process. However, with the increase of network information, the ACO algorithm faces various constraints such as local optimization and feature redundancy for selecting optimal pathways (Peng et al. 2018).

#### Differential evaluation optimization

The differential evolution (DEV) algorithm is renowned for its simplicity and powerful stochastic direct search method. Besides, DEV has proven an efficient and effective method for searching global optimal solutions for the multimodal objective function, utilizing N-D-dimensional parameter vectors (Seme and Štumberger 2011). It does not require a specific starting point, and it operates effectively on a population candidate solution. The constant value N denotes the population; in every module, a new generation solution is determined and compared with the previous generation of the population member. It is a repetition process and runs until it reaches the maximum number of generations (i.e., G_max_). The $G$ defines the generation number of populations which can be written in mathematical proportional order. If the initial population vector is $S_{G}$, then $S_{G} = i_{1,G,} i_{2,G} \ldots \ldots ,i_{NP,G,}$ and $G = 0, \ldots ,G_{max}$

$$i_{n, G} ,n = 1,2, \ldots \ldots ..,N$$

The initial population $S_{G = 0}$ is generated using random within given boundaries, which can be written in the following equation:

16 $$i_{j,0}^{n} = rand_{j} \left[ {0,1} \right]\left( {i_{j}^{\left( U \right)} - i_{j}^{\left( L \right)} } \right) + i_{j}^{\left( L \right)} ,n = 1,2, \ldots ,NP,j = 1,2, \ldots ,D$$

where $rand_{j}$[0, 1] is the uniformly distributed number at the interval [0,1], which is chosen a new for each $j$. $D$ represents the boundary condition. In contrast, $\left( U \right)$ and $\left( L \right)$ represents the upper and lower limit of the boundary vector parameters. For every generation, a new random vector is randomly created, selecting vectors from the previous generation from the following manner:

17 $$c_{j,G}^{n} = \left\{ {\begin{array}{*{20}l} {i_{j,G - 1}^{r} + F\left( {i_{j,G - 1}^{r} - i_{j,G - 1}^{r} } \right)} \hfill & {\quad if\;rand_{j} \left[ {0,1} \right] \le } \hfill \\ {i_{j,G - 1}^{n} } \hfill & { \quad otherwise } \hfill \\ \end{array} } \right\}$$

where $r$ is the number of optimizations, $c$ is the candidate vector, $CR \in \left[ {0,1} \right]$ and $F \in \left[ {0,2} \right]$ control parameter. $k$ is the randomly selected index that ensures the difference between the candidate vector and the generation vector. The population for new the new generation $S_{G}$ will be assembled from the vector of the previous generation $S_{G - 1}$ and the candidate vectors $c_{j,G}^{n}$ the following equation can describe selection:

18 $$\begin{aligned} & G = 0, \ldots ,G_{max} ;\;\;n = 1,2, \ldots \ldots ..,NP \\ & I_{G}^{n} = \left\{ {\begin{array}{*{20}l} {c_{G}^{n} } \hfill & {\quad if f\left( {c_{G}^{n} } \right) \le f(I_{G - 1}^{n} )} \hfill \\ {I_{G - 1}^{n} } \hfill & { \quad otherwise} \hfill \\ \end{array} } \right\} \\ \end{aligned}$$

The process repeats with the following generation population number until it satisfies the pre-defined objective function.

#### Particle swarm optimization

The particle swarm optimization (PSO) method was developed for continuous non-linear functions optimization having roots in artificial life and evolutionary computation (Kennedy and Eberhart 1995). The method was constructed using a simple concept that tracks each particle’s current position in the swarm by implementing a velocity vector for particle’s previous to the new position. However, the movement of the particles in the swarm depends on the individuals’ external behavior. Therefore, the process is very speculative, uses each particle’s memory to calculate a new position and gain knowledge by the swarm as a whole. Nearest neighbor velocity matching and craziness, eliminating ancillary variables, and incorporating multidimensional search and acceleration by distance were the precursor of PSO algorithm simulation (Eberhart and Shi 2001). Each particle in the simulation coordinates in the n-dimensional space calculation and responds to the two quality factors called ‘*gbest*’ and ‘*pbest*’. *gbest* represents the best location and value of particle in the population globally, and *pbest* represents the best-fitted solution achieved by the particle so far in the population swarm. Thus, at each step in the swarm, the PSO concept stands, each particle changing its acceleration towards its two best quality factor locations. The acceleration process begins by separating random numbers and presenting the optimal ‘gbest’ and ‘pbest’ locations. The basic steps for the PSO algorithm are given below, according to (Eberhart and Shi 2001):

1. The process starts with initializing sample random particles with random velocities and locations on n-dimensions in the design space.
2. The velocity vector for each particle in the swarm is carried out in the next step as the initial velocity vector value.
3. Plot the velocity vector value and compare particle fitness evaluation with particle’s *pbest*. If the new value is better than the initial value, update the new velocity vector value as *pbest* and previous location equal to the current location in the design space.
4. This step compares the fitness evaluation with the particles’ overall previous global best. If the current value is better than *gbest,* update it to a new *gbest* value and location*.*
5. The velocity and position of the particle can be changed according to the equations:
  19 $$v_{nd} = v_{nd} + m_{1} *rand\left( x \right)*\left( {p_{nd} - z_{nd} } \right) + m_{2} *Rand\left( x \right)*\left( {p_{gd} - z_{nd} } \right)$$
  20 $$z_{nd} = z_{nd} + v_{nd}$$
6. Repeat step 2 and continue until the sufficiently fitted value and position are achieved.

Particle swarm optimization is well known for its simple operative steps and performance for optimizing a wide range of functions. PSO algorithm can successfully solve the design problem with many local minima and deal with regular and irregular design space problems locally and globally. Although PSO can solve problems more accurately than other traditional gradient-based optimizers, the computational cost is higher in PSO (Ventor and Sobieszczanski-Sobieski 2003).

#### Genetic algorithm

The genetic algorithm (GA) is a heuristic search method based on natural selection and evolution principles and concepts. This method was introduced by John Holland in the mid-seventies, inspired by Darwin’s theory of descent with modification by natural selection. To determine the optimal set of parameters, GA mimics the reproduction behavior of the biological populations in nature. It has been proven effective for solving cutting-edge optimization problems in the selection process. It can also handle regular and irregular variables, non-traditional data partitioning, and non-linear objective functions without requiring gradient information (Hassan et al. 2004). The basic steps for the PSO algorithm are given below:

- The determination of the maximum outcomes from an objective function, the genetic algorithm uses the following function:
  21 $$f = f\left( {\left( {y_{1} + y_{2} } \right), \ldots .\left( {y_{n} + y_{n + 1} } \right)_{n} } \right)$$
  where $$n$$ is the number of decision variables $$y_{i} \in \left[ {y_{i}^{min} ,y_{i}^{max} } \right]$$ with a discretization step $$\delta y_{i}$$. The initial boundary conditions $$y_{i}^{min} ,y_{i}^{max}$$ determined at the beginning of the simulation. $$\delta y_{i}$$ is the determines the physical parameters $$y_{i}$$ performances in the experiment. These decision variables are represented by a sequence of binary digits ($$GENES)$$.
- The decisions variables are given within initial boundary conditions $y_{i} = y_{i}^{min} + \left( {GENE i} \right)* \delta y_{i}$, where $GENE i \in \left[ {0,2^{{n_{i} }} - 1} \right]$ refers to the value of GENES. $n_{i}$ is the bit length of each GENE, which is the first integer where $y_{i}^{min} + 2^{{n_{i} }} - 1*\delta y_{i} \ge y_{i}^{max}$. The total number of bits in each DNA refers $n_{sum} = \sum\nolimits_{i = 1}^{n} {n_{i} }$. The algorithm process starts with a random selection of objectives. After evaluation of each objective in the fitness function $f = f\left( {\left( {y_{1} + y_{2} } \right), \ldots .\left( {y_{n} + y_{n + 1} } \right)_{n} } \right)$, and rank them from best to worst.

The genetic similarity determines the selection progress indicator. These random individual objectives with rank are transferred to the next generation. The remaining individuals participate in the steps of selection, crossover, and mutation. The individual objective parent selection process can happen several times, and this can be achieved by many different schemes, such as the roulette-wheel ranked method. For any pair of objective parents’ selection, crossover, and mutation process of next generation is defined. After that, the fitness $f$ of all individuals scheduled for the next generation is evaluated. This process repeats from generation to generation until a termination criterion is met.

GA methodology is quite like another stochastic searching algorithm PSO. Both methods begin their search from a randomly generated population of designs that evolve over successive generations. They do not require any specific starting point for the simulation. The first operator is the “selection” procedure similar to the “Survival for the Fittest” principle. The second operator is the “Crossover” operator, mimicking mating in a biological population. Both methods use the same convergence criteria for selecting the optimal solution in the problem space (Hassan et al. 2004). However, GA differs in two ways from the most traditional optimization methods. First, GA does not operate directly on the design parameter vector, but a symbolic parameter known as a chromosome. Second, it optimizes the whole design chromosomes at once, unlike other optimization methods single chromosome at a time (Weile and Michielssen 1997).

#### Complete ensemble empirical mode decomposition with adaptive noise (CEEMDAN)

The complete ensemble empirical mode decomposition with adaptive noise (CEEMDAN) decomposition approach is initiated by discretizing the *n*-length predictors of any model χ(*t*) into IMFs (intrinsic model functions) and residues to conform with tolerability. However, to ensure no information leakage in the IMFs and residues, the decomposition is performed separately by training and testing subsets. The actual IMF is produced by taking the empirical mode decomposition (EMD)-grounded IMFs across a trial and combining white noise to model the predictor-target variables. The CEEMDAN is used in machinery, electricity, and medicine such as impact signal denoising, daily peak load forecasting, health degradation monitoring for rolling bearings, friction signal denoising combined with mutual information (Li et al. 2019).

The CEEMDAN process is as follows:

*Step 1* The decomposition of *p*-realizations of $\chi \left[ n \right] = \varepsilon_{1} \omega^{p} \left[ n \right]$ using EMD to develop their first intrinsic approach, as explained according to the equation:

23 $$\widehat{{IMF_{1} }}\left[ n \right] = \frac{1}{p}\mathop \sum \limits_{p = 1}^{P} IMF_{1}^{p} \left[ n \right] = I\overline{M}F_{1} \left[ n \right]$$

*Step 2* Putting *k* = 1, the 1st residue is computed following Eq. (1).

24 $$Res_{1} \left[ n \right] = \chi \left[ n \right] - \widehat{{IMF_{1} }}\left[ n \right]$$

*Step 3* Putting *k* = 2, the 2nd residual is obtained as

25 $$\widehat{{IMF_{2} }}\left[ n \right] = \frac{1}{p}\mathop \sum \limits_{p = 1}^{P} E_{1} (r_{1} \left[ n \right] + \varepsilon_{1} E_{1} \left( {\omega^{p} \left[ n \right]} \right))$$

*Step 4* Setting *k* = 2… *K* calculates the *k*th residue as.

26 $$Res_{k} \left[ n \right] = Res_{k - 1} \left[ n \right] - \widehat{{IMF_{k} }}\left[ n \right]$$

*Step 5* Now, we decompose the realizations $Res_{k} \left[ n \right] + \varepsilon_{1} E_{1} \left( {\omega^{p} \left[ n \right]} \right), {\text{Here}}, k = 1, \ldots K$ until their first model of EMD is reached; Here the (*k* + 1) is

27 $$\widehat{IMF}_{{\left( {k + 1} \right)}} \left[ n \right] = \frac{1}{p}\mathop \sum \limits_{p = 1}^{P} E_{1} (r_{k} \left[ n \right] + \varepsilon_{k} E_{k} \left( {\omega^{p} \left[ n \right]} \right))$$

*Step 6* Now, the *k* value is incremented, and steps 4–6 are repeated. Consequently, the final residue is achieved:

28 $$RES_{k} \left[ n \right] = \chi \left[ n \right] - \mathop \sum \limits_{k = 1}^{K} \widehat{{IMF_{k} }}$$

Here, *K* is the limiting case (*i.e*., the highest number of modes). To comply with the replicability of the earliest input, $\chi \left[ n \right].,$ the following is performed for the CEEMDAN approach.

29 $$\chi \left[ n \right] = \mathop \sum \limits_{k = 1}^{K} \widehat{{IMF_{k} }} + RES_{k} \left[ n \right]$$

### Model implementation procedure

It is crucial to optimize the objective model’s architecture to incorporate the relationship between predictors and model. A multi-phase CNN-GRU and GRU model is built using Python-based deep learning packages such as *TensorFlow* and *Keras*. A total of nine statistical metrics was used to investigate the forecasting robustness of the models that have been integrated. An Intel i7 powered the model with a 3.6 GHz processor and 16 GB of memory. Deep learning libraries like *Keras* (Brownlee 2016; Ketkar 2017) and *TensorFlow* (Abadi et al. 2016) were used to demonstrate algorithms for the proposed models. In addition, packages like *matplotlib* (Barrett et al. 2005) and *seaborn* (Waskom 2021) were used for visualization.

**Fig. 3:**
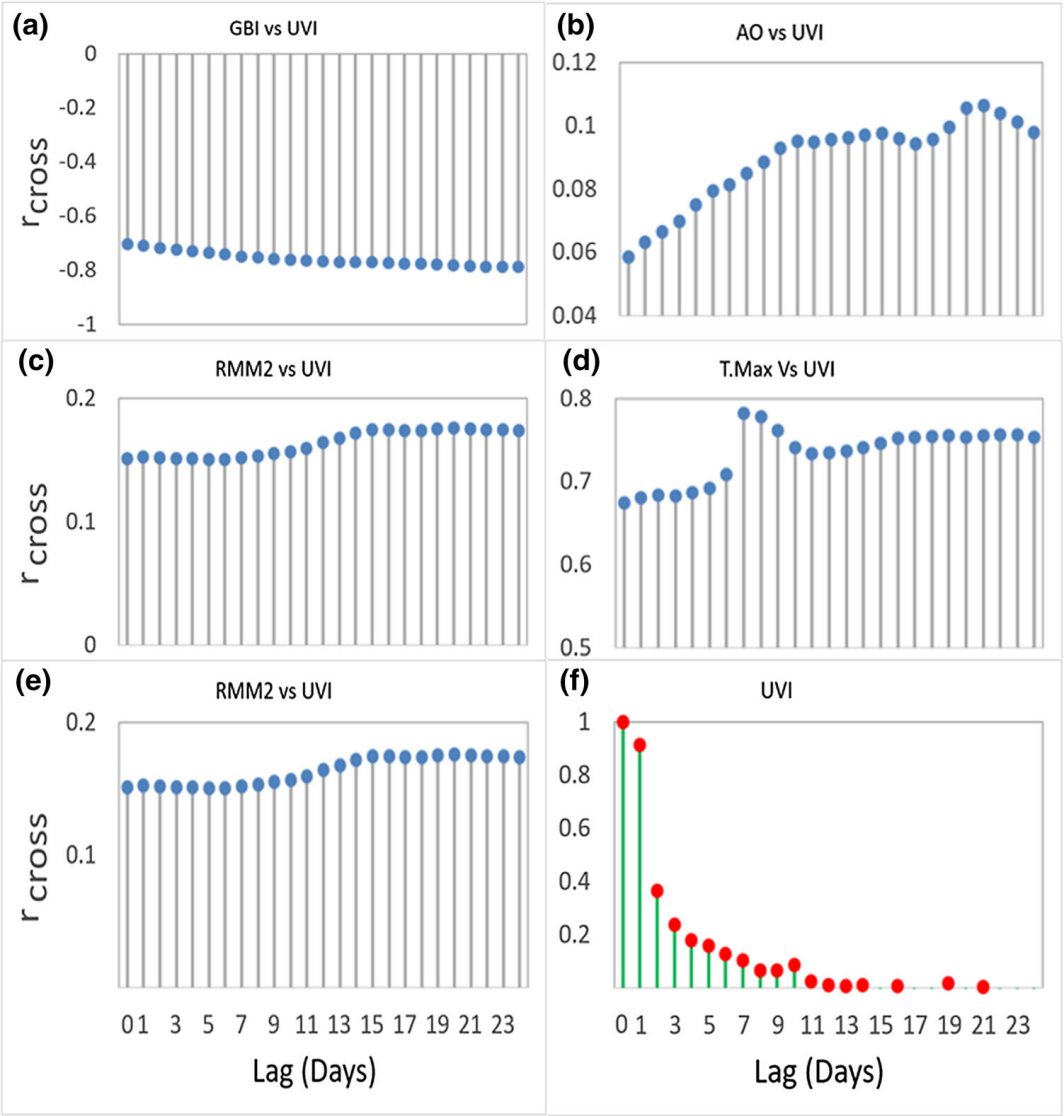
**a**–**e** Correlogram showing the covariance between the objective variable (UVI) and the predictor variables in terms of the Cross-correlation coefficient (r_cross_) and **f** Partial autocorrelation function (PACF) plot of the UVI time series exploring the antecedent behavior in terms of the lag of UVI every day

**Fig. 4:**
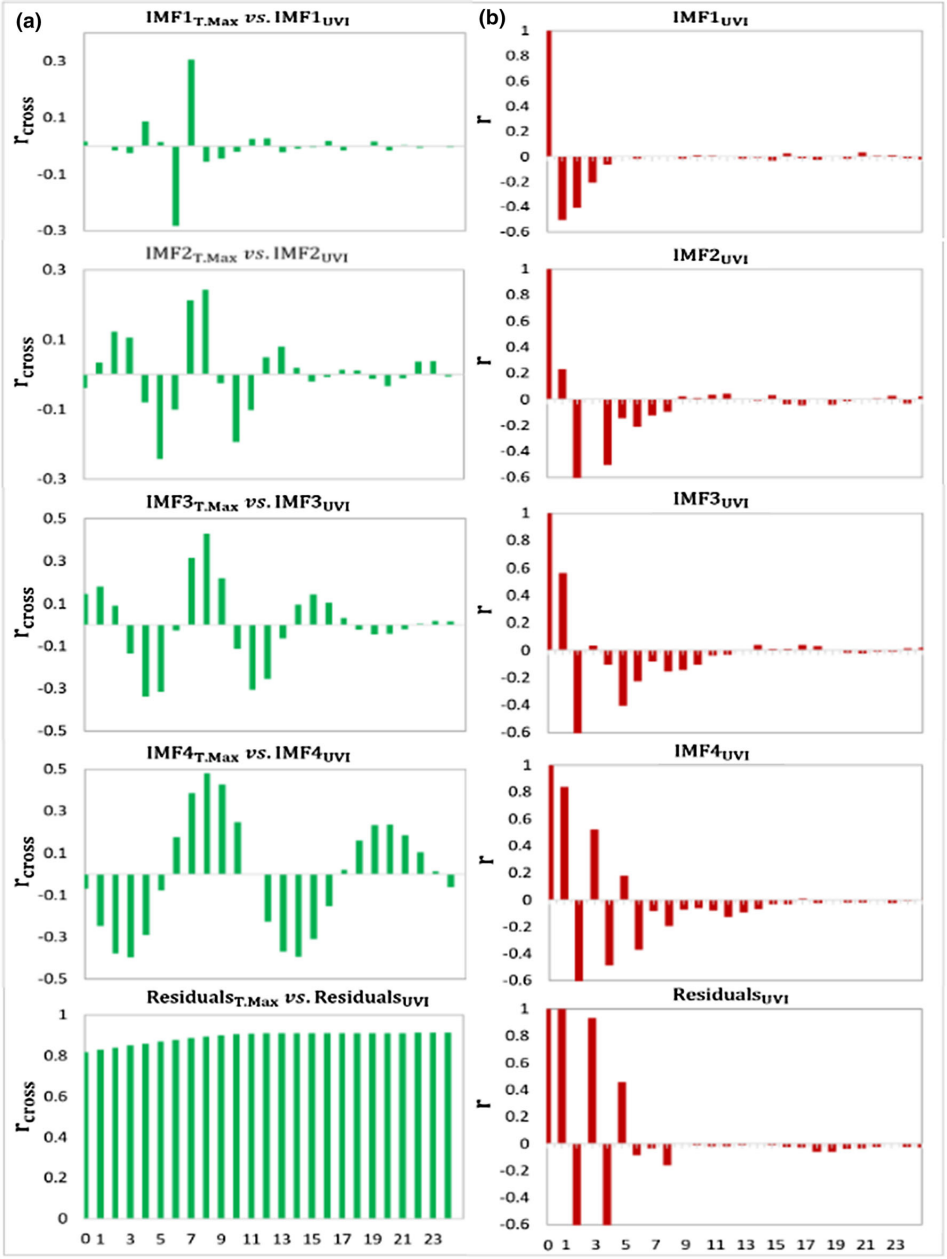
**a** Correlogram showing the covariance between the objective variable (UVI) and the CEEMDAN decomposed T.Max (IMF1_T.Max_ to Residuals_T.Max_) in terms of the Cross-correlation coefficient (r_cross_) and **b** Partial autocorrelation function (PACF) plot of the CEEMDAN decomposed UVI time series exploring the antecedent behavior in terms of the lag of UVI every day

The determination of the model’s valid predictors does not have any precise formula. However, the literature suggests three methods, i.e., trial-and-error, the autocorrelation function (ACF), partial autocorrelation function (PACF), and the cross-correlation function (CCF) approaches, for selecting lagged UVI memories and predictors to make an optimal model. In this study, the PACF was used to determine significant antecedent behavior in terms of the lag of UVI (Tiwari and Adamowski 2013; Tiwari and Chatterjee 2010). Figures 3f and 4b demonstrated the PACF for UVI time series showing the antecedent behavior in terms of the lag of UVI and decomposed UVI (i.e., IMF_n_) where antecedent daily delays are apparent. Generally, the CCF selects the input signal pattern based on the predictors’ antecedent lag (Adamowski et al. 2012). The CCF determined the predictors’ statistical similarity to the target variable (Figs. 3a–e, 4a). A set of significant input combinations was selected after evaluating each predictor’s *r*_*cross*_ with UVI. The figure shows that the highest correlation between predictor variables and UVI was found for all stations at lag zero (i.e., *r*_*cross*_ = 0.22 – 0.75). AOD and GBI demonstrated significant *r*_*cross*_ from 0.65 to 0.80 and 0.68 to 0.75, respectively. Some predictors with insignificant lags such as AO, CT, and OTC were also considered to increase the predictors’ diversity. The CCF with UVI with predictors significantly varied for all other stations. However, selecting lags from the cross-correlation function is identical to the objective stations.

**Fig. 5:**
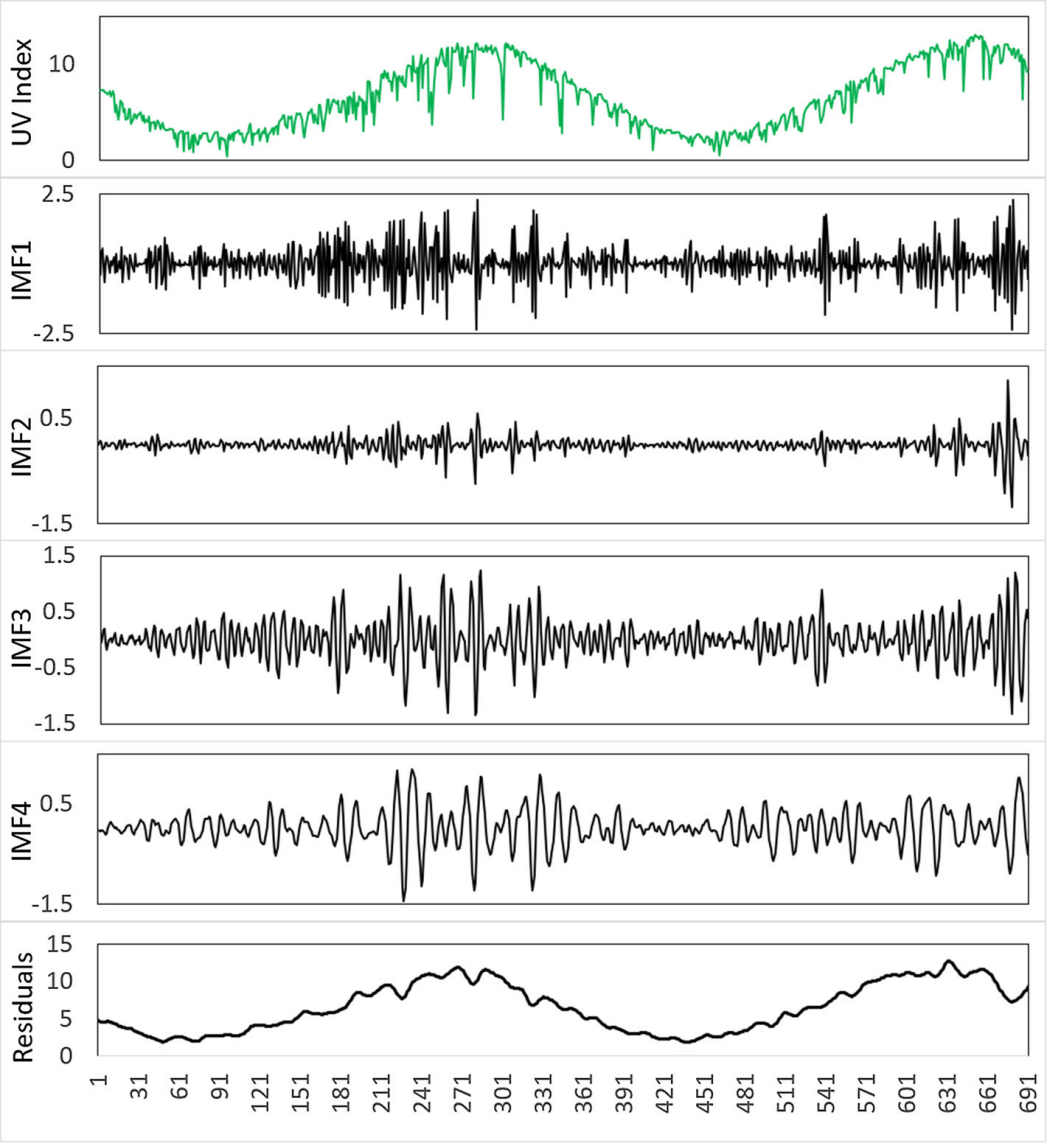
An example time-series showing data features in IMFs and residuals produced by the CEEMDAN transformation of daily maximum UV Index for the case of Perth study site

As mentioned, the CEEMDAN method was used to decompose the data sets. The daily time series of UVI data and predictor variables were decomposed into respective daily IMFs and a residual component using CEEMDAN procedures. The example of the IMFs and the residual component of the respective CEEMDAN is shown in Fig. 5. PACF was applied to the daily IMFs and residual component time series generated above. An individual input matrix was created for each IMF, and the residual component was made up based on the significant lagged memory with that of IMF of target UVI. These separate input matrices were used to forecast future IMFs and residual components. Next, the anticipated IMFs and residuals were combined to produce daily forecasts of UVI values. Note that the CEEMDAN transformations are completely self-adaptive and data-dependent multi-resolution techniques. As such, the number of IMFs and the residual component generated are contingent on the nature of the data.

The predictor variables were used to forecast the UVI were normalized between 0 and 1 to minimize the scaling effect of different variables as follows:

30 $$U_{norm} = \frac{{U - U_{min} }}{{U_{max} - U_{min} }}$$

In Eq. (30), $U$ is the respective predictors, $U_{min}$ is the minimum value for the predictors, $U_{max}$ is the maximum value of the data and $U_{norm}$ is the normalized value of the data. After normalizing the predictor variables, the data sets were partitioned. To build predictive models, input data must be divided into training, testing, and validation sets. The training set is used to train the model, then used to learn more about the data over time. This validation technique tries to provide information for modifying model hyperparameters. Separate from training sets, validation sets are used to examine and validate models. After training a model, the test set is frequently used to evaluate it. This study uses the first 70% of the data sets for training, the middle 15% were used for testing, and the remaining 15% of the data sets were considered validation data.

The CLSTM model was followed by developing a hybrid LSTM model with 3-layered CNN and 4-layered LSTM, as illustrated in Fig. 2. The traditional antecedent lagged matrix of the daily predictors’ variables was applied using the conventional models. The prior application of the optimization algorithm was made before using CCF and PACF and before significant predictors were removed from the model. The theoretical details of CNN and LSTM are already given in Sect. 2. Based on a trial-and-error approach, the hyper-parameters (as stated in Table 4 located in the “Appendix”) for all respective models. The computational complexity cost associated with the learning procedure of ML models is a significant concern; this cost is inversely proportional to the size of the dataset used for training and the algorithm used for hyperparameter selection, and it is directly related to the dataset size used for training (Ghimire et al. 2019). This time-consuming process requires a grid search for the optimal parameters for each model. For example, the search for each model takes approximately 10–11 h. After finding the optimal parameters, the computational time for training and testing becomes significantly less (< 10 min), as shown in Fig. 2. Using a pooling layer to control overfitting issues in the training phase, the CLSTM hybrid predictive model may be made smaller and more controlled, reducing the number of parameters and computation required the network. All the flattening layer outputs are routed to the respective inputs of the LSTM recurrent layer, which is routed to the final output of the flattening layer. Table 2 shows the selected predictors using four optimization techniques associated with the UVI, and the optimal parameters of four feature selection algorithms are tabulated in Table 5 from the “Appendix”.

**Table 2:**
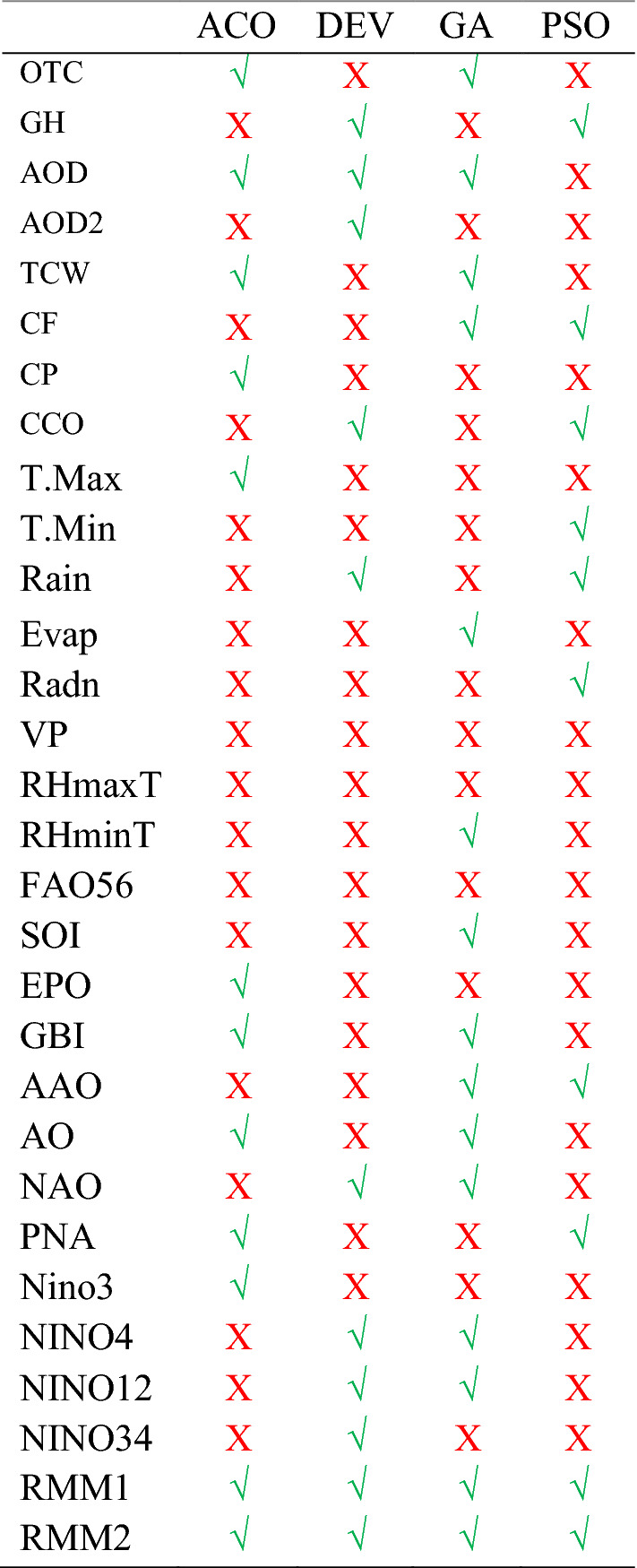
List of selected input variables prior applying in the proposed model using four optimization techniques (i.e., ACO, DEV, GA and PSO)

### Model performance assessment

In this study, the effectiveness of the deep learning hybrid model was assessed using a variety of performance evaluation criteria, e.g., Pearson’s Correlation Coefficient (*r*), root mean square error (*RMSE*), Nash–Sutcliffe efficiency (*NSE*) (Nash and Sutcliffe 1970), and mean absolute error (*MAE*). The relative *RMSE (*denoted as *RRMSE)* and relative *MAE (*denoted as *RMAE)* were used to explore the geographic differences between the study stations.

The exactness of the relationship between predicted and observed values were used to evaluate a predictive model's effectiveness. When the error distribution in the tested data is Gaussian, the root means square error (RMSE) is a more appropriate measure of model performance than the mean absolute error (MAE) (Chai and Draxler 2014), but for an improved model evaluation, the Legates-McCabe’s (*LM*) Index is used as a more sophisticated and compelling measure (Legates and McCabe 2013; Willmott et al. 2012). Mathematically, the metrics are as follows:

1. Correlation coefficient (*r*):
  31 $$r = { }\left\{ {\frac{{\mathop \sum \nolimits_{i = 1}^{N} \left( {UVI_{obs} - \overline{UVI}_{obs} } \right)\left( {UVI_{for} - \overline{UVI}_{for} } \right)}}{{\sqrt {\mathop \sum \nolimits_{i = 1}^{N} \left( {UVI_{obs} - \overline{UVI}_{obs} } \right)^{2} \mathop \sum \nolimits_{i = 1}^{N} \left( {UVI_{for} - \overline{UVI}_{for} } \right)^{2} } }}} \right\}^{2}$$
2. Mean absolute error (*MAE*):
  32 $${\text{MAE}} = \frac{1}{{\text{N}}}\mathop \sum \limits_{{{\text{i}} = 1}}^{{\text{N}}} \left| {UVI_{{{\text{for}} - }} UVI_{{{\text{obs}}}} } \right|$$
3. Root mean squared error (*RMSE*):
  33 $${\text{RMSE}} = \sqrt {\frac{1}{{\text{N}}}\mathop \sum \limits_{{{\text{i}} = 1}}^{{\text{N}}} \left( {{\text{UVI}}_{{{\text{for }} - }} UVI_{{{\text{obs}}}} } \right)^{2} }$$
4. Nash–Sutcliffe Efficiency (*NS*):
  34 $${\text{NSE}} = 1 - { }\left[ {1 - { }\frac{{\mathop \sum \nolimits_{{{\text{i}} = 1}}^{{\text{N}}} (UVI_{for} )^{2} }}{{\mathop \sum \nolimits_{{{\text{i}} = 1}}^{{\text{N}}} \left( {UVI_{obs} - { }\overline{UVI}_{for} } \right)^{2} }}} \right])$$
5. Legates–McCabe’s Index (*LM*):
  35 $$LM = 1 - \left[ {\frac{{\mathop \sum \nolimits_{{{\text{i}} = 1}}^{{\text{N}}} \left| {UVI_{{{\text{for}} - }} UVI_{{{\text{obs}}}} } \right|}}{{\mathop \sum \nolimits_{{{\text{i}} = 1}}^{{\text{N}}} \left| {\left| {UVI_{{{\text{obs}} - }} \overline{UVI}_{{{\text{obs}}}} } \right|} \right|}}} \right]$$
6. Relative Root Mean Squared Error (*RRMSE*, %):
  36 $$RRMSE\left( \% \right) = \frac{{\sqrt {\frac{1}{N}\mathop \sum \nolimits_{{{\text{i}} = 1}}^{{\text{N}}} \left( {UVI_{{{\text{for}} - }} UVI_{{{\text{obs}}}} } \right)^{2} } }}{{\frac{1}{N}\mathop \sum \nolimits_{i = 1}^{N} (UVI_{obs} )}} \times 100$$
7. Relative Mean Absolute Error (RMAE*,* %):
  37 $$RMAE \left( \% \right) = \frac{{\frac{1}{{\text{N}}}\mathop \sum \nolimits_{{{\text{i}} = 1}}^{{\text{N}}} \left| {UVI_{{{\text{for }} - }} UVI_{{{\text{obs}}}} } \right|}}{{\frac{1}{N}\mathop \sum \nolimits_{i = 1}^{N} (UVI_{obs} )}} \times 100$$

In Eqs. (31–37), $UVI_{obs}$ and $UVI_{{{\text{for}}}}$ represents the observed and forecasted values for *i*th test value; $\overline{UVI}_{obs}$ and $\overline{UVI}_{for}$ refer to their averages, accordingly, and *N* is defined as the number of observations, while the *CV* stands for the coefficient of variation.

## Results

This section describes results obtained from the proposed hybrid deep learning model (i.e., CEEMDAN-CLSTM) and other hybrid models (i.e., CEEMDAN-CGRU, CEEMDAN-LSTM, CEEMDAN-GRU, CEEMDAN-DT, CGRU, and CLSTM), and the standalone LSTM, GRU, DT, MLP, and SVR models. Four feature selection algorithms (i.e., ACO, DEV, GA, and PSO) were incorporated to obtain the optimum features in model building. Seven statistical metrics from Eqs. (31)–(37) were used to analyze the models in the testing dataset and visual plots to justify the forecasted results’ justification.

The hybrid deep learning model (i.e., CEEMDAN-CLSTM) demonstrated high r and NS values and low RMSE and MAE compared to their standalone models (Table 3). The best overall performance was recorded using the CEEMDAN-CLSTM model with the Genetic Algorithm with the highest correlation (i.e., r = 0.996), the highest data variance explained (i.e., NS = 0.997), and the lowest errors (i.e., RMSE = 0.162 and MAE = 0.119). The performance was followed by the same model with PSO (i.e., r ≈ 0.996; NS ≈ 0.992; RMSE ≈ 0.216; MAE ≈ 0.163) and ACO (i.e., r ≈ 0.996; NS ≈ 0.993; RMSE ≈ 0.220; MAE ≈ 0.165). The single deep learning models (i.e., LSTM and GRU) performed better than the single machine learning models (i.e., DT, SVR, and MLP). Moreover, the hybrid deep learning models without a CNN (i.e., CEEMDAN-GRU and CEEMDAN-GRU) also demonstrated higher forecasting accuracy (i.e., r = 0.973 – 0.993; RMSE = 0.387 – 0.796) in comparison with standalone deep learning models (i.e., r ≈ 0.959 – 0.981; RMSE ≈ 0.690 – 0.986). The following models’ performance is then predicted by the CNN-GRU, CEEMDAN-GRU, and GRU models in that order.

**Table 3:**
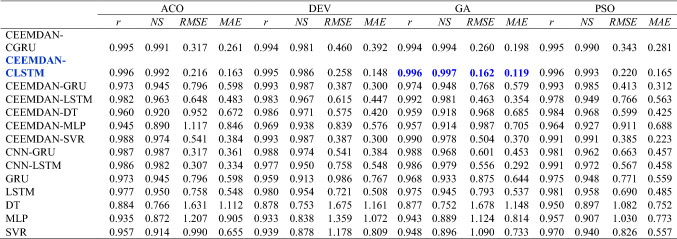
Evaluation of hybrid CEEMDAN-CLSTM *vs*. benchmark (CNN-GRU, CNN-LSTM, CEEMDAN-GRU, CEEMDAN-LSTM, GRU and LSTM) models for Perth observation sites

The correlation coefficient (*r*), root mean square error (*RMSE*), mean absolute error (*MAE*) and Nash–Sutcliffe coefficient (*NS*) are computed between forecasted and observed *UVI* for 7 Day ahead periods in testing phase. The optimal model is boldfaced (blue)

**Fig. 6:**
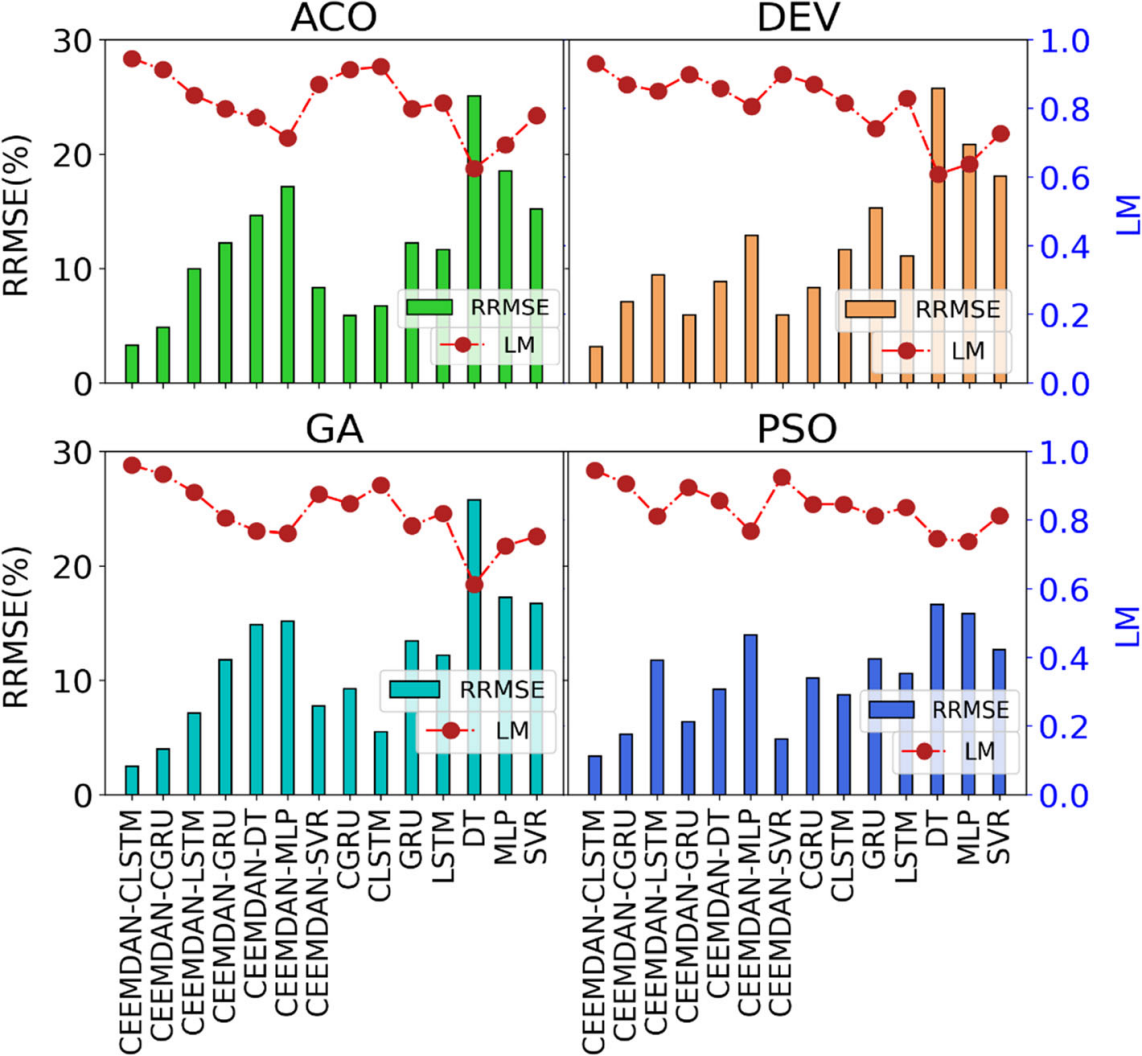
Comparison of the forecasting skill for all proposed models in terms of the relative error: RRMSE (%) and Legate McCabe Index (LM) within the testing period

RRMSE and LM for all tested models were used to assess the robustness of the proposed hybrid models and for comparisons. The magnitude of RRMSE (%) and LM for the objective model (CEEMDAN-CLSTM) shown in Fig. 6 indicates that the proposed hybrid model performed significantly better than other benchmark models. The RRMSE and LM values ranged between 2 and 3.5% and between 0.982 and 0.991, respectively. The performance indices (i.e., RRMSE and LM) using four optimization algorithms were higher for the CEEMDAN-CGRU model. Overall, the CEEMDAN-CLSTM model with the GA optimization methods provided the best performance (i.e., RRMSE =  ~ 2.0%; LM = 0.991), indicating its high efficiency in forecasting the future UV-Index a higher degree of accuracy.

**Fig. 7:**
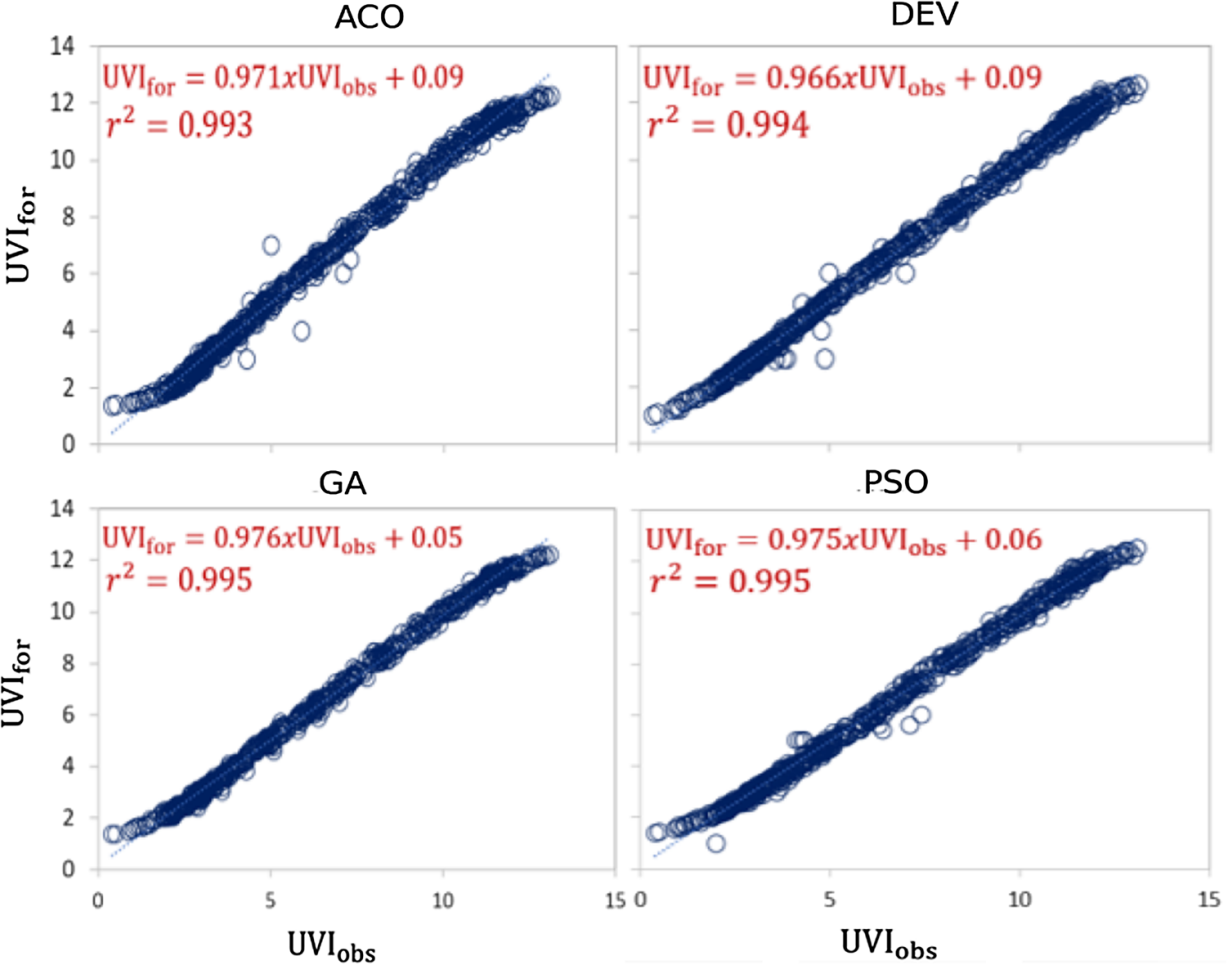
Scatter plot of forecasted with observed UVI (UVI) of Perth station CEEMDAN-CLSTM model. A least square regression line and coefficient of determination (R^2^) with a linear fit equation are shown in each sub-panel

A precise comparison of forecasted and observed UVI can also be seen by examining the scatterplot of forecasted (UVI_for_) and observed (UVI_obs_) UVI for four optimization algorithms (i.e., ACO, PSO, DEV, and GA) (Fig. 7). Here, scatter plots showed the coefficient of determination (r^2^) and a least-square fitting line, along with the equation for UVI and an observed UVI close to the forecasted UVI. As demonstrated in Fig. 7, it also appears that the proposed hybrid model performed better when compared with other applied models. However, among the four optimization techniques applied, the hybrid deep learning model (i.e., CEEMDAN-CLSTM) optimized with the GA outperformed the other models in forecasting the UVI. The hybrid CEEMDAN-CLSTM model calculated magnitudes from the GA, which came the closest to unity, with an m|r^2^ of 0.976|0.995 in pairs. The performance is followed by ACO and DEV algorithms with a potential pair (ACO: 0.975|0.995; DEV: 0.966|0.994). The outliers (i.e., the extremes) are closer to the fitted line, while the y-intercept (i.e., the starting point) is approximately 0.05 units away from zero (0) using the GA method. The other models had outliers, resulting in their intercepts deviating from the ideal value. In conclusion, the CEEMDAN-CLSTM model performed the best for the GA.

**Fig. 8:**
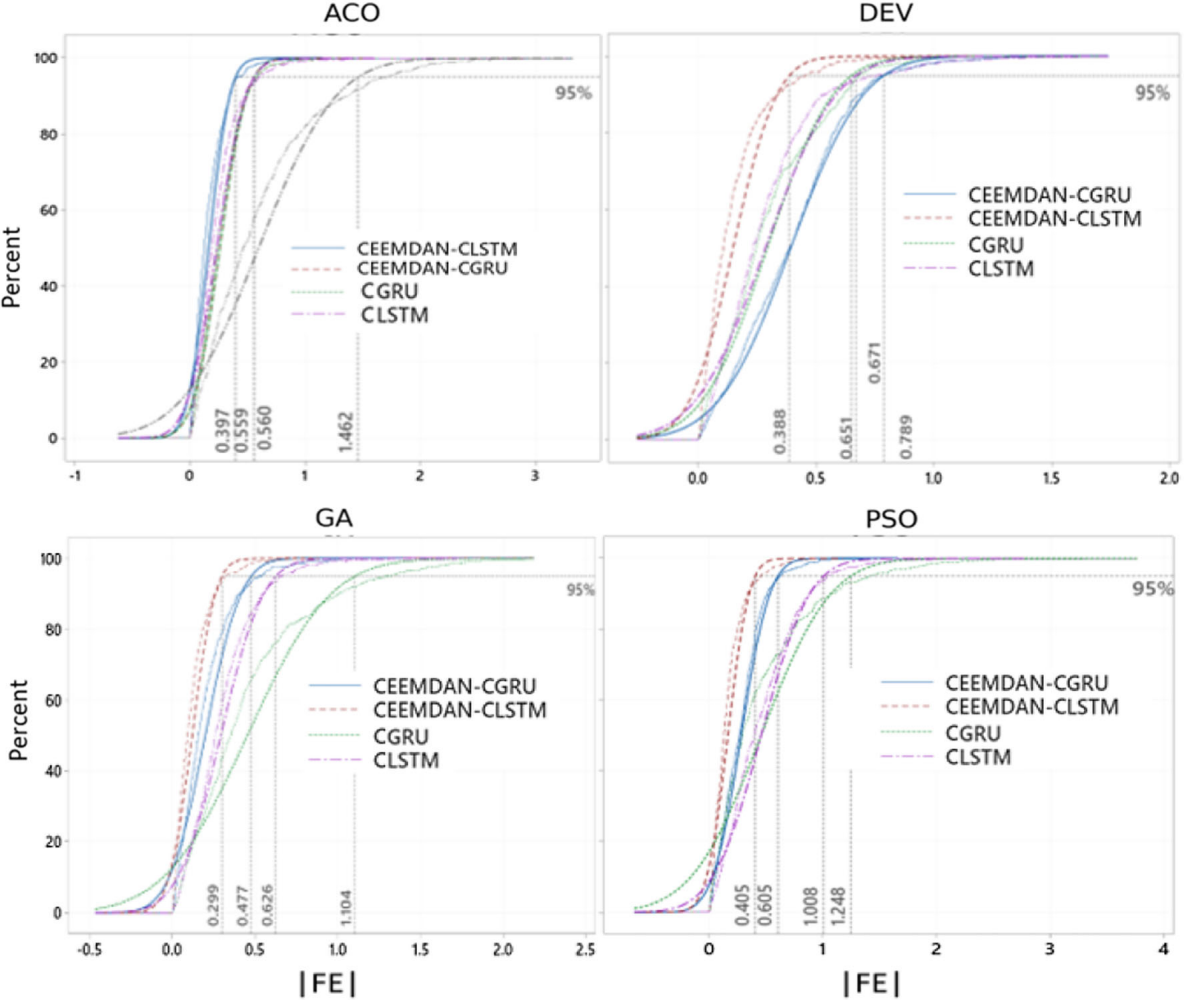
Empirical cumulative distribution function (CDF) in forecasting error |FE| for CEEMDAN-CGRU, CEEMDAN-CLSTM, CNN-GRU, and CNN-LSTM model, shown for the 95 percentile on ECDF

**Fig. 9:**
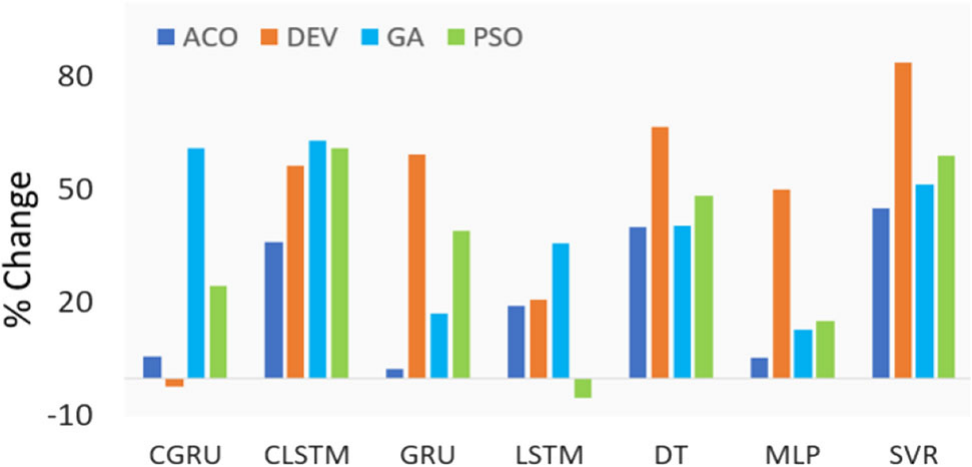
Effect on percent change (%) of RMAE using CEEMDAN as a feature extraction approach in forecasting UVI at Perth station using Genetic Algorithm (GA), Ant Colony Optimization (ACO), Particle Swarm Optimization (PSO), and Differential Evolution (DEV)

The proposed hybrid deep learning model (i.e., CEEMDAN-CLSTM) was further assessed employing the ECDFs of absolute forecast error (|FE|) (Fig. 8). Total 95% forecasted values using the CEEMDAN-CLSTM model with GA demonstrated a small error ranging between 0.01 and 0.299, with a substantially larger error for the CCGRU model (i.e., 0.477), followed by the CLSTM model (i.e., 0.626) and CGRU (i.e., 1.104). For the other optimization algorithms, nearly the same level of performance was observed. Predictions ranging between the 95th and 98th percentile were preferred over objective models, which performed best in the current forecast. However, Fig. 9 showed the effect of applying CEEMDAN as a feature extraction method on the percent change in RMAE values within the testing phase of UVI forecast incrementally. The contribution of the data decomposition method (i.e., CEEMDAN) was significant in the model implementation. The increment of RMAE in percent using GA was found between 17 to 63%, whereas the CLSTM showed the highest percentage of decrement (i.e., 63%). Moreover, the PSO optimized model showed that the *RMAE* (%) values with the deep learning model appeared to decrease by ~ 2 to 60%, and the lowest decreasing RMAE was found for the ACO algorithm with a reduction of ~ 3% to 36%. However, the CLSTM model using four optimization methods showed the highest improvement among all the deep learning approaches that reduced the RMSE from 36 to 63%. It is worth mentioning that the percent increase in *RMAE* was ~ 83% for the DEV algorithm using the SVR method. Overall, the CEEMDAN, as a data decomposition algorithm for UVI forecasting with four optimization algorithms, showed significant improvement over the testing phase.

**Fig. 10:**
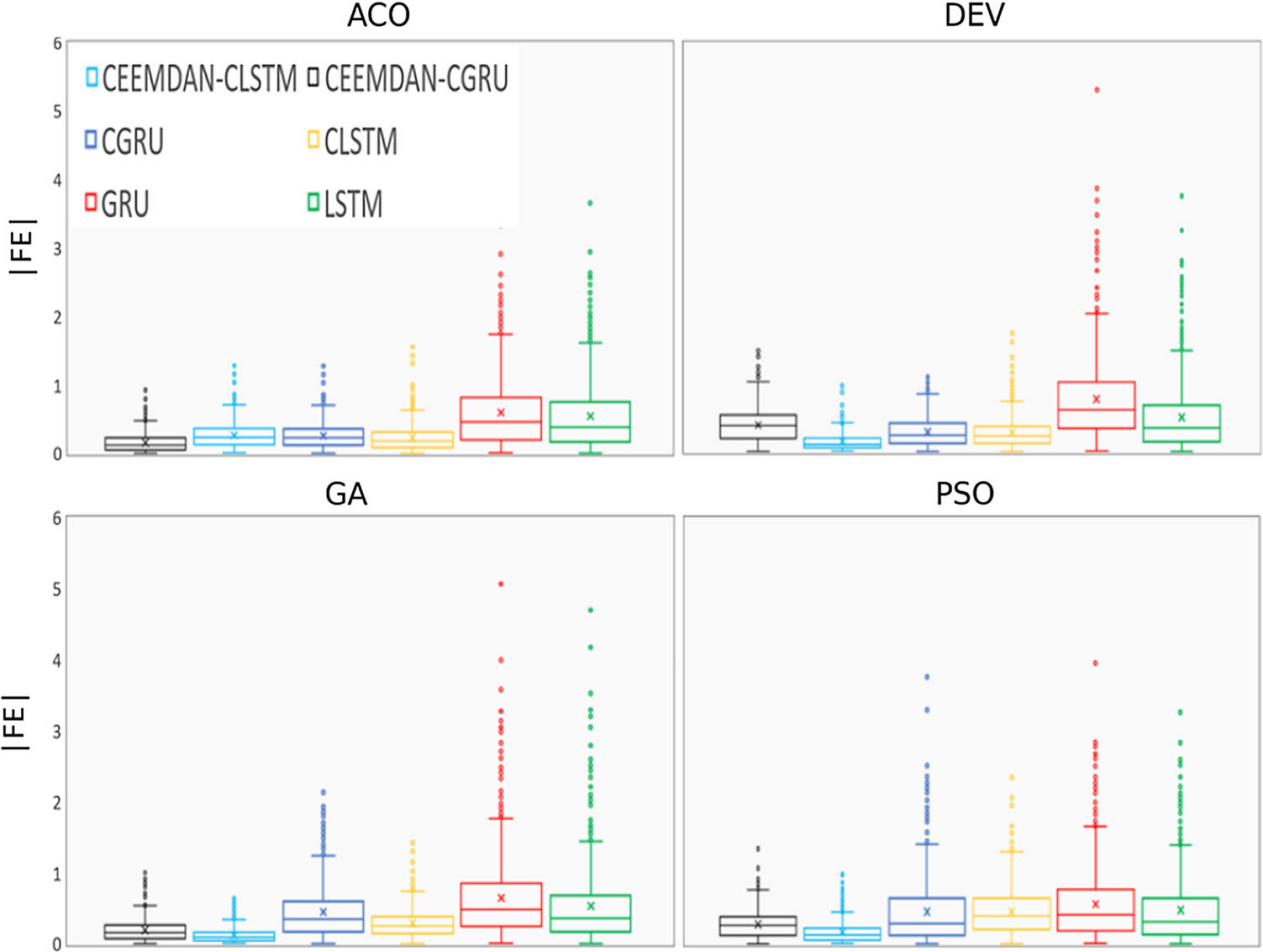
Evaluation of the performance of the proposed hybrid deep learning, CEEMDAN-CLSTM model with the comparative benchmark models based on the absolute forecasted error |FE| using four optimization techniques

After additional analysis, the forecasted-to-observed UVI and absolute forecasting errors are displayed in Fig. 10. The absolute forecasted error has a maximum dispersion of (|FE| =|UVI_for_ – UVI_obs_|). The box plot demonstrated the data dispersal of the observed and forecasted UVI from the proposed deep learning approaches and other comparing models. Figure 10 provides a clear visualization of the data concerning quartiles distinctly outliers. The lower end of the plot lies between the lower quartile (25th percentile) and upper quartile (75^th^ percentile). It is evident that the median of the forecasted and the observed UVI for the CEEMDAN-CLSTM model with the GA optimization. Moreover, the DEV-based CEEMDAN-CLSTM model showed identical forecasting to the GA-based CEEMDAN-CLSTM model with a slight variation. A more in-depth inspection of the absolute forecasted error (|FE|) from the hybrid CEEMDAN-CLSTM model for two optimizations (i.e., GA and DEV) further strengthens the suitability of the hybrid CLSTM approach in forecasting the UVI of Perth station of Australia with the narrowest distribution in comparison with other models. A significant percentage (98%) of the |FE| in the first error brackets (0 <|FE|< 0.15) was observed for the GA-based CEEMDAN-CLSTM model, while for the DEV-based model, the percentage is 95%.

**Fig. 11:**
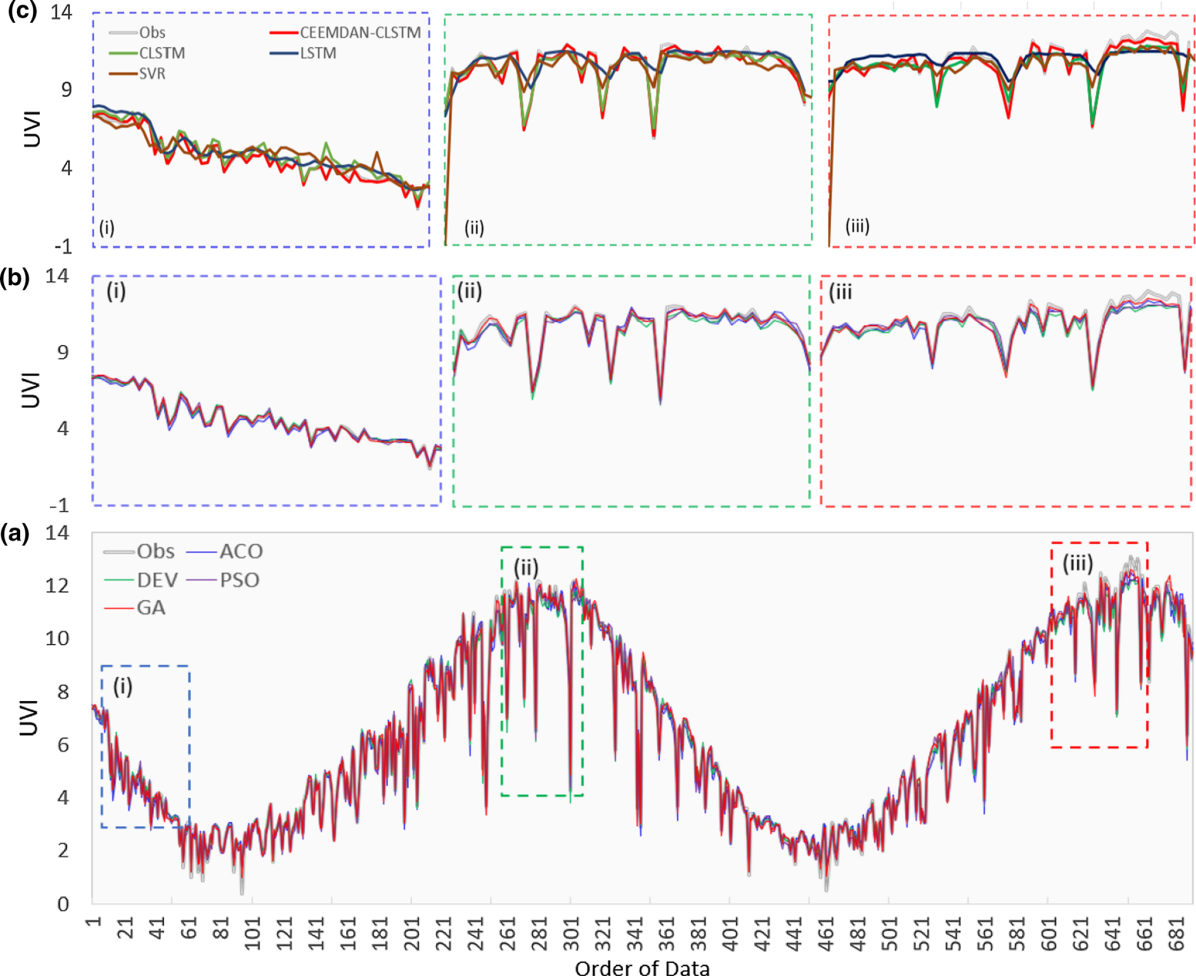
Time series of daily maximum UV index (UVI) for observed UVI and forecasted UVI for the objective model, CEEMDAN-CLSTM using four optimization approaches

With the help of a time series plot, we can better understand forecasting ability and refine the proposed model, taking it from standalone to hybrid model. The time series plot of forecasted and observed UVI using CEEMDAN-CLSTM optimized by four optimization methods is depicted in Fig. 11. The results showed that the proposed GA-based CEEMDAN-CLSTM model is close to the observed UVI, indicating that the model has high predictive accuracy. The application of the GA in the model optimization resulted in a significant improvement in forecasted UVI. For other algorithms that use the CEEMDAN-CLSTM model, it is discovered that the forecasted UVI is accurate when compared to the other optimization methods.

**Fig. 12:**
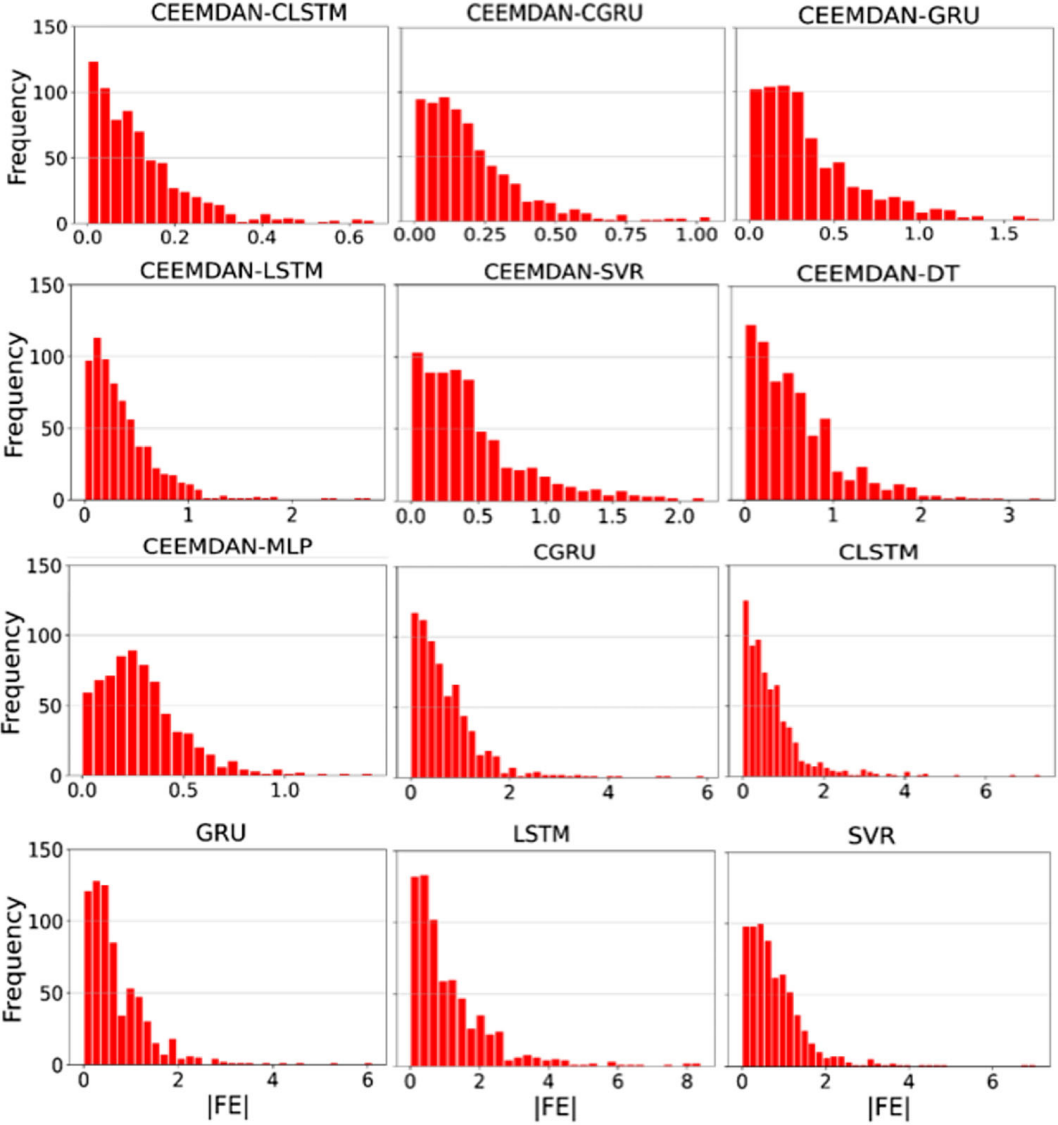
Illustration of the frequency of absolute value of estimation errors (|EE|) of the proposed hybrid deep learning CEEMDAN-CLSTM model and comparing models using Genetic Algorithm (GA)

Finally, Fig. 12 presents a comprehensive interpretation by illustrating the absolute forecasting error frequency distributions (|FE|) using all GA-based models for Perth stations of Australia. It is apparent from Fig. 12 that the CEEMDAN-CLSTM model provided significantly improved distributions with the maximum 98% forecasting error (|FE|) within the first error brackets (0 <|FE|< 0.10). It is also noteworthy that the CEEMDAN-CGRU model showed a higher percentage of |FE| between 0 and 0.25 of all forecasting yielded a considerably small error and the remaining 15% of simultaneously produced forecasting error between 0.25 and 1.0. The highest forecasted error was found for machine learning models when all models’ |FE| value (i.e., SVR, MLP, and DT) was considered.

## Discussion

The establishment of robust predictive modelling of the UV index and physical interpretation is critical for various practical applications, such as helping policymakers in their daily health impact assessment. These systems emulate how a human expert would solve a complex forecasting problem by reasoning through a set of UVI-related predictors rather than through conventional or procedural methods. These methods warrant continuous irradiance measurement or radiative transfer models, which are tedious (as discussed in the introduction) and often inaccurate. This study demonstrated the efficacy of hybrid deep learning methods in forecasting UVI on a near real-time horizon. The study site was in Perth, Western Australia, Australia, where skin cancer is significantly high. An accurate forecasting system in this region is therefore essential.

To function effectively, alert systems must generate accurate irradiance forecasts. Still, UVI is generally determined by many factors (i.e., the solar zenith, altitude, cloud fraction, aerosol and optical properties, albedo, and vertical ozone profile) (Deo et al. 2017). The study extensively utilized four optimization techniques (i.e., GA, ACO, DEV, and PSO) to have optimum predictors used in UVI forecasting as tabulated in Table 2. The incorporated predictors from three distinct data sets (i.e., SILO, MODIS, and CI) were optimized. The optimization techniques selected a diversified list of variables except for RMM1 and RMM2, as four algorithms selected them both. The predictors like ozone total column, AOD, and cloud fraction were significant using the GA algorithm. In most cases, the hydro-meteorological variables were insignificant by all four algorithms that agree with UV concentration’s general concept. The objective algorithm (i.e., GA) selected SOI, GBI, AAO, Nino4, Nino12, RMM1, and RMM2 as potential predictors as well. The ground-based measurements and modelling studies are essential (Alados et al. 2004, 2007) but are challenging to implement in practice. Furthermore, secondary factors affecting UV levels (i.e., clouds or aerosols) are rarely known with sufficient precision. Considering the practical feasibility, an algorithm that is data-efficient, simple to develop, flexible, and user-friendly should be considered a viable alternative for information (Igoe et al. 2013a, b; Parisi et al. 2016). Therefore, our developed forecasting model will play a vital role in adopting prompt measures without difficulties.

This study shows significant improvement from the previous studies in forecasting UVI in Australia. Deo et al. (2017) applied machine learning techniques to predict the UVI in Australia, demonstrating a substantial performance. However, this study found improved forecasting in a 7-day ahead time horizon by integrating three distinct types of datasets. The study can be further extended to other parts of Australia and around the world to develop an early warning framework of solar radiation UV index for better management and mitigation of UV-related health hazards.

The proposed hybrid deep learning network (i.e., CEEMDAN-CLSTM) for predicting surface UV radiation also demonstrated low errors in forecasting, i.e., showing around 10% error for the next-day forecast and 13–16% error for 7-day up to the 4-week forecast. This further affirms that the quantitative UV forecast is appropriate for heliotherapy applications, which tolerates up to 10–25% error levels. The CEEMDAN-CLSTM’s performance is competitive on UV data from multiple regions. Thus, the CEEMDAN-CLTSM model can be adapted to forecast other beneficial UV action spectra, such as vitamin D production and erythemal UV index. A fundamental limitation of machine learning is its overfitting tendency on the training dataset and often does not generalize well to other datasets from different distributions. In the context of UV forecasting, this dictates that the model must be retrained with data from the weather station to be used for that geographic region. In a geographical region with the highly variable weather condition, such as London in 2019, artificial neural network models’ performance dropped significantly (Raksasat et al. 2021). This capability of the model to extract seasonal patterns may also explain why the addition of ozone, cloud fraction, and AOD information significantly improved the performance of CEEMDAN-CLSTM, particularly when the GA algorithm was applied.

## Conclusion

This study conducted a daily UV Index forecasting at Perth station using aggregated significant antecedent satellite-driven variables associated with UV irradiance. The forecasting was made using a novel hybrid deep learning model (i.e., CEEMDAN-CLSTM) and compared with other benchmark models such as LSTM, GRU, DT, SVR, etc. Four optimization methods were employed to extract the crucial features of the response variable (i.e., the UVI). After applying the proposed model and benchmarked models, the model’s merits were evaluated using different statistical metrics, graphical plots, and relevant discussions. The key findings are summarized as follows:

- The CEEMDAN-CLSTM hybrid model demonstrated excellent forecasting ability compared to its counterpart models.
- The GA optimization algorithm is appeared to be an attractive option for selecting mechanistically meaningful features of the dependable variable compared to the other three optimization techniques.
- The performance metrics showed that the GA and CEEMDAN-optimized models had better performance and higher efficiency metrics (i.e., r, NS, and LM) and lower error metrics (i.e., MSE and RMSE).
- However, in UVI forecasting, the standalone models’ (i.e., LSTM, GRU, DT, and SVR) performances were poor compared to the proposed hybrid model.

Adapted to an Australian climate in the sub-tropics during peak summer-time conditions, applying a CLSTM model to forecast the UVI is a novel deep learning approach. The forecasts derived from our data were within one UVI unit of the actual measured values indicating the remarkable forecasting capability. Therefore, this data-driven model would be of tremendous help for the decision-makers to promptly protect public health without delay. It has the tremendous potential to be adopted by a more significant segment of the community, particularly children and the elderly facing a greater risk of developing skin cancer (i.e., melanoma) in the Australian region and worldwide.

## Appendix

See Tables

**Table 4:** The optimum hyper-parameter of the CLSTM model

Model	Model hyper-parameter Names	Search space for optimal hyper-parameters
*Optimally selected hyper-parameters*		
CLSTM	Convolution Layer 1 (C1)	40
	C1- Activation function	Relu
	C1-Pooling Size	1
	Convolution Layer 2 (C2)	20
	C2- Activation function	Tanh
	C2-Pooling Size	1
	Convolution Layer 3 (C3)	50
	C3- Activation function	Relu
	C3-Pooling Size	1
	LSTM Layer 1 (L1)	100
	L1- Activation function	Relu
	LSTM Layer 2 (L2)	80
	L2- Activation function	ReLU
	LSTM Layer 3 (L4)	100
	L3- Activation function	Relu
	LSTM Layer 4 (L4)	50
	L4- Activation function	ReLU
	Drop-out rate	0.2
	Optimiser	SDG
	Learning Rate	0.001
	Padding	Same
	Batch Size	5
	Epochs	1000

^4^ and

**Table 5:** The optimum characteristics of four feature selection algorithms (i.e., GA, ACO, PSO, and DEV)

Characteristics	Optimal value
*Genetic algorithm (GA)*	
Number of chromosomes	10
Maximum number of generations	100
Crossover rate	0.8
Mutation rates	0.3
*Ant colony optimization (ACO)*	
Number of ants	10
Maximum number of iterations	100
Coefficient control tau	1
Coefficient control eta	2
Initial tau	1
Initial beta	1
Pheromone	0.2
Coefficient	0.5
*Differential evolution (DEV)*	
Number of vectors	14
Maximum number of generations	100
Crossover rate	0.9
*Particle Swarm Optimization (PSO)*	
Number of particles	10
Maximum number of iterations	150
Cognitive factor	2
Social factor	2
Maximum velocity	6
Maximum bound on inertia weight	0.9
Minimum bound on inertia weight	0.4

^5^.

## Abbreviations

ACO: Ant colony optimization
ACF: Autocorrelation function
ANFIS: Adaptive neuro-fuzzy inference system
ANN: Artificial neural network
AO: Arctic oscillation
ARPANSA: Australian radiation protection and nuclear safety agency
BCC: Basal cell carcinoma
BOM: Bureau of meteorology
CEEMDAN: Complete ensemble empirical mode decomposition with adaptive noise
CEEMDAN-CLSTM: Hybrid model integrating the CEEMDAN and CNN algorithm with LSTM
CEEMDAN-CGRU: Hybrid model integrating the CEEMDAN and CNN algorithm with GRU
CEEMDAN-GRU: Hybrid model integrating the CEEMDAN algorithm with GRU
CNN-LSTM (or CLSTM): Hybrid model integrating the CNN algorithm with LSTM
CNN-GRU (or CGRU): Hybrid model integrating the CNN algorithm with GRU
CEEMDAN: Complete ensemble empirical mode decomposition with Adaptive Noise
CEEMDAN-DT: Hybrid model integrating the CEEMDAN algorithm with DT
CEEMDAN-MLP: Hybrid model integrating the CEEMDAN algorithm with MLP
CEEMDAN-SVR: Hybrid model integrating the CEEMDAN algorithm with SVR
CNN: Convolutional neural network
COVID-19: Coronavirus disease 2019
CCF: Cross-correlation function
EEMD: Ensemble empirical mode decomposition
EMD: Empirical mode decomposition
DEV: Differential evolution
DL: Deep learning
DT: Decision tree
DWT: Discrete wavelet transformation
ECDF: Empirical cumulative distribution function
ELM: Extreme learning machine
EMI: El-Nino southern oscillation Modoki indices
ENSO: El Niño Southern Oscillation
FE: Forecasting error
GA: Genetic algorithm
GB: Giga bite
GIOVANNI: Geospatial online interactive visualization and analysis infrastructure
GRU: Gated recurrent unit
GLDAS: Global land data assimilation system
GSFC: Goddard space flight centre
IMF: Intrinsic mode functions
LM: Legates-McCabe’s index
LSTM: Long- short term memory
MAE: Mean absolute error
MAPE: Mean absolute percentage error
MARS: Multivariate adaptive regression splines
MDB: Murray–Darling basin
MJO: Madden–Julian oscillation
ML: Machine learning
MLP: Multi-layer perceptron
MODWT: Maximum overlap discrete wavelet transformation
MODIS: Moderate resolution imaging spectroradiometer
MRA: Multi-resolution analysis
MSE: Mean squared error
NAO: North Atlantic oscillation
NASA: National aeronautics and space administration
NCEP: National centers for environmental prediction
NO: Nitrogen oxide
NOAA: National oceanic and atmospheric administration
NMSC: Non-melanoma skin cancer
NSE: Nash–Sutcliffe efficiency
PACF: Partial autocorrelation function
PDO: Pacific decadal oscillation
PNA: Pacific North American
PSO: Particle swarm optimization
r: Correlation coefficient
RMM: Real-time multivariate MJO series
GA: Genetic algorithm
BRF: Boruta random forest
RMSE: Root-mean-square-error
RNN: Recurrent neural network
RRMSE: Relative root-mean-square error
SAM: Southern annular mode
SARS-CoV-2: Severe acute respiratory syndrome Coronavirus 2
SCC: Squamous cell carcinoma
SILO: Scientific information for landowners
SOI: Southern oscillation index
SST: Sea surface temperature
SVR: Support vector regression
US: United States
UV: Ultraviolet
UVI: Ultraviolet index
WHO: World Health Organization
WI: Willmott’s index of agreement

## References

[CR1] Abadi M, Barham P, Chen J, Chen Z, Davis A, Dean J, Devin M, Ghemawat S, Irving G, Isard M, Kudlur M, Levenberg J, Monga R, Moore S, Murray DG, Steiner B, Tucker P, Vasudevan V, Warden P, Wicke M, Yu Y, Zheng X (2016) TensorFlow: a system for large-scale machine learning. Presented at the 12th {USENIX} symposium on operating systems design and implementation ({OSDI} 16), pp 265–283

[CR2] Adamowski J, Chan HF, Prasher SO, Ozga-Zielinski B, Sliusarieva A (2012). Comparison of multiple linear and non-linear regression, autoregressive integrated moving average, artificial neural network, and wavelet artificial neural network methods for urban water demand forecasting in Montreal, Canada. Water Resour Res.

[CR3] Ahmed MH, Lin L-S (2021). Dissolved oxygen concentration predictions for running waters with different land use land cover using a quantile regression forest machine learning technique. J Hydrol.

[CR4] Ahmed AAM, Deo RC, Ghahramani A, Raj N, Feng Q, Yin Z, Yang L (2021). LSTM integrated with Boruta-random forest optimiser for soil moisture estimation under RCP4.5 and RCP8.5 global warming scenarios. Stoch Environ Res Risk Assess.

[CR5] Ahmed A, Deo RC, Feng Q, Ghahramani A, Raj N, Yin Z, Yang L (2021). Hybrid deep learning method for a week-ahead evapotranspiration forecasting. Stoch Environ Res Risk Assess.

[CR6] Ahmed A, Deo RC, Raj N, Ghahramani A, Feng Q, Yin Z, Yang L (2021). Deep learning forecasts of soil moisture: convolutional neural network and gated recurrent unit models coupled with satellite-derived MODIS, observations and synoptic-scale climate index data. Remote Sens.

[CR7] Ahmed AM, Deo RC, Feng Q, Ghahramani A, Raj N, Yin Z, Yang L (2021). Deep learning hybrid model with Boruta-Random forest optimiser algorithm for streamflow forecasting with climate mode indices, rainfall, and periodicity. J Hydrol.

[CR8] Alados I, Mellado JA, Ramos F, Alados-Arboledas L (2004). Estimating UV erythemal irradiance by means of neural networks. Photochem Photobiol.

[CR9] Alados I, Gomera MA, Foyo-Moreno I, Alados-Arboledas L (2007). Neural network for the estimation of UV erythemal irradiance using solar broadband irradiance. Int J Climatol.

[CR10] Alfadda A, Rahman S, Pipattanasomporn M (2018). Solar irradiance forecast using aerosols measurements: a data driven approach. Sol Energy.

[CR11] Allaart M, van Weele M, Fortuin P, Kelder H (2004). An empirical model to predict the UV-index based on solar zenith angles and total ozone. Meteorol Appl.

[CR12] Anderiesz C, Elwood M, Hill DJ (2006). Cancer control policy in Australia. Aust N Z Health Policy.

[CR13] Antanasijević D, Pocajt V, Perić-Grujić A, Ristić M (2014). Modelling of dissolved oxygen in the Danube River using artificial neural networks and Monte Carlo Simulation uncertainty analysis. J Hydrol.

[CR14] ARPANSA (2021) Australian radiation and nuclear protection agency 2021 realtime UV levels [WWW Document]. ARPANSA. https://www.arpansa.gov.au/our-services/monitoring/ultraviolet-radiation-monitoring/ultraviolet-radiation-index. Accessed 9 July 2021

[CR15] Barrett P, Hunter J, Miller JT, Hsu J-C, Greenfield P (2005) matplotlib: a portable python plotting package 347, 91

[CR16] Baumgaertner AJG, Seppälä A, Jöckel P, Clilverd MA (2011). Geomagnetic activity related NO_x_ enhancements and polar surface air temperature variability in a chemistry climate model: modulation of the NAM index. Atmos Chem Phys.

[CR17] Beltrán-Castro J, Valencia-Aguirre J, Orozco-Alzate M, Castellanos-Domínguez G, Travieso-González CM (2013) Rainfall forecasting based on ensemble empirical mode decomposition and neural networks. In: International work-conference on artificial neural networks. Springer, pp 471–480

[CR18] BOM (2020) Australia’s official weather forecasts & weather radar: Bureau of Meteorology [WWW Document]. http://www.bom.gov.au/. Accessed 9 July 2021

[CR19] Boniol M, Ringborg U, Brandberg Y, Breitbart E, Greinert R (2016). Descriptive epidemiology of skin cancer incidence and mortality. Skin cancer prevention.

[CR20] Brownlee J (2016) Deep learning with python: develop deep learning models on Theano and tensor flow using Keras. Machine Learning Mastery

[CR21] Chai T, Draxler RR (2014). Root mean square error (RMSE) or mean absolute error (MAE)? Arguments against avoiding RMSE in the literature. Geosc Model Dev.

[CR22] Chen JP, Yang L, Wang LK, Zhang B, Wang LK, Hung Y-T, Shammas NK (2006). Ultraviolet radiation for disinfection. Advanced physicochemical treatment processes, handbook of environmental engineering.

[CR23] Chen C, Jiang H, Zhang Y, Wang Y (2010) Investigating spatial and temporal characteristics of harmful Algal Bloom areas in the East China Sea using a fast and flexible method. In: 2010 18th international conference on geoinformatics. Presented at the 2010 18th international conference on geoinformatics, pp 1–4. 10.1109/GEOINFORMATICS.2010.5567490

[CR24] Christian O, Volkmar CM, Adnana P-G, van Faassen Ernst E, Christian H, Malte K, Daniel H, Manfred M, Norbert P, Suschek CV (2009). Whole body UVA irradiation lowers systemic blood pressure by release of nitric oxide from intracutaneous photolabile nitric oxide derivates. Circ Res.

[CR25] Deo RC, Downs N, Parisi AV, Adamowski JF, Quilty JM (2017). Very short-term reactive forecasting of the solar ultraviolet index using an extreme learning machine integrated with the solar zenith angle. Environ Res.

[CR26] Dey R, Salem FM (2017) Gate-variants of Gated Recurrent Unit (GRU) neural networks. In: 2017 IEEE 60th international midwest symposium on circuits and systems (MWSCAS). Presented at the 2017 IEEE 60th international midwest symposium on circuits and systems (MWSCAS), pp 1597–1600. 10.1109/MWSCAS.2017.8053243

[CR27] Di C, Yang X, Wang X (2014). A four-stage hybrid model for hydrological time series forecasting. PLoS ONE.

[CR28] Downs N, Butler H, Parisi A (2016). Solar ultraviolet attenuation during the Australian (Red Dawn) dust event of 23 September 2009. Bull Am Meteorol Soc.

[CR29] Eberhart, Shi Y (2001) Particle swarm optimization: developments, applications and resources. In: Proceedings of the 2001 congress on evolutionary computation (IEEE Cat. No.01TH8546). Presented at the proceedings of the 2001 congress on evolutionary computation (IEEE Cat. No.01TH8546), vol 1, pp 81–86. 10.1109/CEC.2001.934374

[CR30] Fan J, Wu L, Ma X, Zhou H, Zhang F (2020). Hybrid support vector machines with heuristic algorithms for prediction of daily diffuse solar radiation in air-polluted regions. Renew Energy.

[CR31] Fernández-Delgado M, Cernadas E, Barro S, Ribeiro J, Neves J (2014). Direct Kernel Perceptron (DKP): Ultra-fast kernel ELM-based classification with non-iterative closed-form weight calculation. Neural Netw.

[CR32] Fouilloy A, Voyant C, Notton G, Motte F, Paoli C, Nivet M-L, Guillot E, Duchaud J-L (2018). Solar irradiation prediction with machine learning: forecasting models selection method depending on weather variability. Energy.

[CR33] Furuhashi T, Torii K, Ikumi K, Kato H, Nishida E, Morita A (2020). Ultraviolet al phototherapy for the treatment of localized scleroderma. J Dermatol.

[CR34] Ghimire S, Deo RC, Downs NJ, Raj N (2019). Global solar radiation prediction by ANN integrated with European Centre for medium range weather forecast fields in solar rich cities of Queensland Australia. J Clean Prod.

[CR35] Giovanni [WWW Document] (2021) https://giovanni.gsfc.nasa.gov/giovanni/. Accessed 9 July 2021

[CR36] Gray NF, Percival SL, Yates MV, Williams DW, Chalmers RM, Gray NF (2014). Chapter thirty-four: ultraviolet disinfection. Microbiology of waterborne diseases.

[CR37] Guo Y, Liu Y, Oerlemans A, Lao S, Wu S, Lew MS (2016). Deep learning for visual understanding: a review. Neurocomput Recent Dev Deep Big Vis.

[CR38] Hassan R, Cohanim B, de Weck O, Venter G (2004) A comparison of particle swarm optimization and the genetic algorithm. In: 46th AIAA/ASME/ASCE/AHS/ASC structures, structural dynamics and materials conference. American Institute of Aeronautics and Astronautics. 10.2514/6.2005-1897

[CR39] Heilingloh CS, Aufderhorst UW, Schipper L, Dittmer U, Witzke O, Yang D, Zheng X, Sutter K, Trilling M, Alt M, Steinmann E, Krawczyk A (2020). Susceptibility of SARS-CoV-2 to UV irradiation. Am J Infect Control.

[CR40] Hendon H, Salby M (1994). The life cycle of the Madden–Julian oscillation. J Atmos Sci.

[CR41] Hochreiter S, Schmidhuber J (1997). Long short-term memory. Neural Comput.

[CR42] Hollaender A, Buy HGD, Ingraham HS, Wheeler SM (1944). Control of air-borne microorganisms by ultraviolet floor irradiation. Science.

[CR43] Huang CJ, Kuo PH (2018). A deep CNN-LSTM model for particulate matter (PM25) forecasting in smart cities. Sensors (Basel).

[CR44] Huang X, Zhang C, Li Q, Tai Y, Gao B, Shi J (2020). A comparison of hour-ahead solar irradiance forecasting models based on LSTM network [WWW document]. Math Probl Eng.

[CR45] Igoe D, Parisi A, Carter B (2013). Smartphones as tools for delivering sun-smart education to students. Teach Sci.

[CR46] Igoe D, Parisi A, Carter B (2013). Characterization of a smartphone camera’s response to ultraviolet A radiation. Photochem Photobiol.

[CR48] Ji X, Shang X, Dahlgren RA, Zhang M (2017). Prediction of dissolved oxygen concentration in hypoxic river systems using support vector machine: a case study of Wen-Rui Tang River, China. Environ Sci Pollut Res.

[CR49] Jiao G, Guo T, Ding Y (2016). A new hybrid forecasting approach applied to hydrological data: a case study on precipitation in Northwestern China. Water.

[CR50] Jiménez-Pérez PF, Mora-López L (2016). Modeling and forecasting hourly global solar radiation using clustering and classification techniques. Sol Energy.

[CR51] Jovanovic B, Collins D, Braganza K, Jakob D, Jones DA (2011). A high-quality monthly total cloud amount dataset for Australia. Clim Change.

[CR52] Juzeniene A, Moan J (2012). Beneficial effects of UV radiation other than via vitamin D production. Dermato-Endocrinology.

[CR53] Kaba K, Kandirmaz HM, Avci M (2017). Estimation of daily sunshine duration using support vector machines. Int J Green Energy.

[CR54] Karimkhani C, Green AC, Nijsten T, Weinstock MA, Dellavalle RP, Naghavi M, Fitzmaurice C (2017). The global burden of melanoma: results from the Global Burden of Disease Study 2015. Br J Dermatol.

[CR55] Kazantzidis A, Smedley A, Kift R, Rimmer J, Berry JL, Rhodes LE, Webb AR (2015). A modeling approach to determine how much UV radiation is available across the UK and Ireland for health risk and benefit studies. Photochem Photobiol Sci.

[CR56] Kennedy J, Eberhart R (1995) Particle swarm optimization. In: Proceedings of ICNN’95: international conference on neural networks. Presented at the Proceedings of ICNN’95: international conference on neural networks, vol 4, pp 1942–1948. 10.1109/ICNN.1995.488968

[CR57] Ketkar N, Ketkar N (2017). Introduction to Keras. Deep learning with python: a hands-on introduction.

[CR58] Kiladis GN, Straub KH, Reid GC, Gage KS (2001). Aspects of interannual and intraseasonal variability of the tropopause and lower stratosphere. Q J R Meteorol Soc.

[CR59] Kroft EBM, Berkhof NJG, van de Kerkhof PCM, Gerritsen RMJP, de Jong EMGJ (2008). Ultraviolet A phototherapy for sclerotic skin diseases: a systematic review. J Am Acad Dermatol.

[CR60] Krzyścin JW, Guzikowski J, Czerwińska A, Lesiak A, Narbutt J, Jarosławski J, Sobolewski PS, Rajewska-Więch B, Wink J (2015). 24 hour forecast of the surface UV for the antipsoriatic heliotherapy in Poland. J Photochem Photobiol B.

[CR61] Latosińska JN, Latosińska M, Bielak J (2015). Towards modelling ultraviolet index in global scale. Artificial neural networks approach. Aerosp Sci Technol.

[CR62] Lau WK-M, Waliser DE (2011). Intraseasonal variability in the atmosphere-ocean climate system.

[CR63] Lecun Y, Bottou L, Bengio Y, Haffner P (1998). Gradient-based learning applied to document recognition. Proc IEEE.

[CR64] Lee S-W, Hwang S-J, Lee S-B, Hwang H-S, Sung H-C (2009). Landscape ecological approach to the relationships of land use patterns in watersheds to water quality characteristics. Landsc Urban Plan.

[CR65] Legates DR, McCabe GJ (2013). A refined index of model performance: a rejoinder. Int J Climatol.

[CR66] Li J, Jiang Y, Xia X, Hu Y (2018). Increase of surface solar irradiance across East China related to changes in aerosol properties during the past decade. Environ Res Lett.

[CR67] Li Y, Chen X, Yu X (2019) Processes | free full-text | a hybrid energy feature extraction approach for ship-radiated noise based on CEEMDAN combined with energy difference and energy entropy [WWW document]. https://www.mdpi.com/2227-9717/7/2/69. Accessed 19 June 2021

[CR68] Liang T, Xie G, Fan S, Meng Z (2020). A combined model based on CEEMDAN, permutation entropy, gated recurrent unit network, and an improved bat algorithm for wind speed forecasting. IEEE Access.

[CR69] Liu Y, Wang L (2021). Drought prediction method based on an improved CEEMDAN-QR-BL model. IEEE Access.

[CR70] Liu H, Tian H, Li Y (2015). Four wind speed multi-step forecasting models using extreme learning machines and signal decomposing algorithms. Energy Convers Manag.

[CR71] Liu B, Wang D, Fu S, Cao W (2017). Estimation of peak flow rates for small drainage areas. Water Resour Manag.

[CR72] Lucas RM, McMichael AJ, Armstrong BK, Smith WT (2008). Estimating the global disease burden due to ultraviolet radiation exposure. Int J Epidemiol.

[CR73] Madden RA, Julian PR (1971). Detection of a 40–50 day oscillation in the Zonal Wind in the Tropical Pacific. J Atmos Sci.

[CR74] Madden RA, Julian PR (1994). Observations of the 40–50-day tropical oscillation: a review. Mon Weather Rev.

[CR75] Mäusezahl D, Christen A, Pacheco GD, Tellez FA, Iriarte M, Zapata ME, Cevallos M, Hattendorf J, Cattaneo MD, Arnold B, Smith TA, Colford JM (2009). Solar drinking water disinfection (SODIS) to reduce childhood diarrhoea in rural Bolivia: a cluster-randomized, controlled trial. PLoS Med.

[CR77] McCarthy WH (2004). The Australian experience in sun protection and screening for melanoma. J Surg Oncol.

[CR78] Mucherino A, Fidanova S, Ganzha M (2015) Ant colony optimization with environment changes: an application to GPS surveying. Presented at the 2015 federated conference on computer science and information systems, pp 495–500. 10.15439/2015F33

[CR79] Nash JE, Sutcliffe JV (1970). River flow forecasting through conceptual models part I: a discussion of principles. J Hydrol.

[CR80] Norval M, Cullen AP, de Gruijl FR, Longstreth J, Takizawa Y, Lucas RM, Noonan FP, van der Leun JC (2007). The effects on human health from stratospheric ozone depletion and its interactions with climate change. Photochem Photobiol Sci.

[CR81] Ouyang Q, Lu W, Xin X, Zhang Y, Cheng W, Yu T (2016). Monthly rainfall forecasting using EEMD-SVR based on phase-space reconstruction. Water Resour Manag.

[CR82] Pak U, Kim C, Ryu U, Sok K, Pak S (2018). A hybrid model based on convolutional neural networks and long short-term memory for ozone concentration prediction. Air Qual Atmos Health.

[CR83] Parisi AV, Downs N, Turner J, Amar A (2016). Online educative activities for solar ultraviolet radiation based on measurements of cloud amount and solar exposures. J Photochem Photobiol, B.

[CR84] Pavlakis KG, Hatzidimitriou D, Drakakis E, Matsoukas C, Fotiadi A, Hatzianastassiou N, Vardavas I (2007). ENSO surface longwave radiation forcing over the tropical Pacific. Atmos Chem Phys.

[CR85] Pavlakis KG, Hatzianastassiou N, Matsoukas C, Fotiadi A, Vardavas I (2008). ENSO surface shortwave radiation forcing over the tropical Pacific. Atmos Chem Phys.

[CR86] Peng H, Ying C, Tan S, Hu B, Sun Z (2018). An improved feature selection algorithm based on ant colony optimization. IEEE Access.

[CR87] Pinker RT, Grodsky S, Zhang B, Chen W (2017). ENSO impact on radiative fluxes as observed from space. J Geophys Res Oceans.

[CR88] Pooi CK, Ng HY (2018). Review of low-cost point-of-use water treatment systems for developing communities. npj Clean Water.

[CR89] Prasad R, Deo RC, Li Y, Maraseni T (2018). Soil moisture forecasting by a hybrid machine learning technique: ELM integrated with ensemble empirical mode decomposition. Geoderma.

[CR90] Prasad R, Ali M, Kwan P, Khan H (2019). Designing a multi-stage multivariate empirical mode decomposition coupled with ant colony optimization and random forest model to forecast monthly solar radiation. Appl Energy.

[CR91] Prasad R, Deo RC, Li Y, Maraseni T (2019). Weekly soil moisture forecasting with multivariate sequential, ensemble empirical mode decomposition and Boruta-random forest hybridizer algorithm approach. CATENA.

[CR92] Pruthi D, Bhardwaj R (2021). Modeling air quality index using optimized neuronal networks inspired by swarms. Environ Eng Res.

[CR93] Qing X, Niu Y (2018). Hourly day-ahead solar irradiance prediction using weather forecasts by LSTM. Energy.

[CR94] Raksasat R, Sri-iesaranusorn P, Pemcharoen J, Laiwarin P, Buntoung S, Janjai S, Boontaveeyuwat E, Asawanonda P, Sriswasdi S, Chuangsuwanich E (2021). Accurate surface ultraviolet radiation forecasting for clinical applications with deep neural network. Sci Rep.

[CR95] Román R, Antón M, Valenzuela A, Gil JE, Lyamani H, Miguel AD, Olmo FJ, Bilbao J, Alados-Arboledas L (2013). Evaluation of the desert dust effects on global, direct and diffuse spectral ultraviolet irradiance. Tellus B Chem Phys Meteorol.

[CR96] Roshan DR, Koc M, Abdallah A, Martin-Pomares L, Isaifan R, Fountoukis C (2020). UV index forecasting under the influence of desert dust: evaluation against surface and satellite-retrieved data. Atmosphere.

[CR97] Saraiya M, Glanz K, Briss PA, Nichols P, White C, Das D, Smith SJ, Tannor B, Hutchinson AB, Wilson KM, Gandhi N, Lee NC, Rimer B, Coates RC, Kerner JF, Hiatt RA, Buffler P, Rochester P (2004). Interventions to prevent skin cancer by reducing exposure to ultraviolet radiation: a systematic review. Am J Prev Med.

[CR98] Seme S, Štumberger G (2011). A novel prediction algorithm for solar angles using solar radiation and differential evolution for dual-axis sun tracking purposes. Sol Energy.

[CR99] Seo Y, Kim S (2016). Hydrological forecasting using hybrid data-driven approach. Am J Appl Sci.

[CR100] Silva CA, Sousa JMC, Runkler TA, Sá da Costa JMG (2009). Distributed supply chain management using ant colony optimization. Eur J Oper Res.

[CR101] Sivamani RK, Crane LA, Dellavalle RP (2009). The benefits and risks of ultraviolet (UV) tanning and its alternatives: the role of prudent sun exposure. Dermatol Clin.

[CR102] Slevin T, Clarkson J, English D (2000). Skin cancer control Western Australia: is it working and what have we learned?. Radiat Prot Dosim.

[CR103] Srivastava R, Tiwari AN, Giri VK (2019). Solar radiation forecasting using MARS, CART, M5, and random forest model: a case study for India. Heliyon.

[CR104] Staiger H, den Outer PN, Bais AF, Feister U, Johnsen B, Vuilleumier L (2008). Hourly resolved cloud modification factors in the ultraviolet. Atmos Chem Phys.

[CR105] Stanton WR, Janda M, Baade PD, Anderson P (2004). Primary prevention of skin cancer: a review of sun protection in Australia and internationally. Health Promot Int.

[CR106] Staples M, Marks R, Giles G (1998). Trends in the incidence of non-melanocytic skin cancer (NMSC) treated in Australia 1985–1995: are primary prevention programs starting to have an effect?. Int J Cancer.

[CR107] Staples MP, Elwood M, Burton RC, Williams JL, Marks R, Giles GG (2006). Non-melanoma skin cancer in Australia: the 2002 national survey and trends since 1985. Med J Aust.

[CR108] Sudhibrabha S, Harold Buchanan Exell R, Sukawat D (2006). Ultraviolet forecasting in Thailand. ScienceAsia.

[CR109] Szenicer A, Fouhey DF, Munoz-Jaramillo A, Wright PJ, Thomas R, Galvez R, Jin M, Cheung MCM (2019). A deep learning virtual instrument for monitoring extreme UV solar spectral irradiance. Sci Adv.

[CR110] Tartaglione N, Toniazzo T, Orsolini Y, Otterå OH (2020). Impact of solar irradiance and geomagnetic activity on polar NOx, ozone and temperature in WACCM simulations. J Atmos Sol Terr Phys.

[CR111] Tian B, Waliser DE, Kahn RA, Li Q, Yung YL, Tyranowski T, Geogdzhayev IV, Mishchenko MI, Torres O, Smirnov A (2008). Does the Madden–Julian oscillation influence aerosol variability?. J Geophys Res Atmos.

[CR112] Timmermann LF, Ritter K, Hillebrandt D, Küpper T (2015). Drinking water treatment with ultraviolet light for travellers: evaluation of a mobile lightweight system. Travel Med Infect Dis.

[CR113] Tiwari MK, Adamowski J (2013). Urban water demand forecasting and uncertainty assessment using ensemble wavelet-bootstrap-neural network models. Water Resour Res.

[CR114] Tiwari MK, Chatterjee C (2010). A new wavelet–bootstrap–ANN hybrid model for daily discharge forecasting. J Hydroinf.

[CR115] Turner EC, Manners J, Morcrette CJ, O’Hagan JB, Smedley ARD (2017). Toward a new UV index diagnostic in the Met Office’s forecast model. J Adv Model Earth Syst.

[CR116] Ventor G, Sobieszczanski-Sobieski J (2003) Particle swarm optimization | AIAA Journal [WWW Document]. 10.2514/2.2111. Accessed 19 June 21

[CR117] Wang F, Yu Y, Zhang Z, Li J, Zhen Z, Li K (2018). Wavelet decomposition and convolutional LSTM networks based improved deep learning model for solar irradiance forecasting. Appl Sci.

[CR118] Wang Y, Yuan Z, Liu H, Xing Z, Ji Y, Li H, Fu Q, Mo C (2022). A new scheme for probabilistic forecasting with an ensemble model based on CEEMDAN and AM-MCMC and its application in precipitation forecasting. Expert Syst Appl.

[CR119] Waskom ML (2021). Seaborn: statistical data visualization. J Open Source Softw.

[CR120] Weile D, Michielssen E (1997). Genetic algorithm optimization applied to electromagnetics: a review. IEEE Trans Antennas Propag.

[CR121] Welch D, Buonanno M, Grilj V, Shuryak I, Crickmore C, Bigelow AW, Randers-Pehrson G, Johnson GW, Brenner DJ (2018). Far-UVC light: a new tool to control the spread of airborne-mediated microbial diseases. Sci Rep.

[CR122] Wells WF, Fair GM (1935). Viability of *B. Coli* exposed to ultra-violet radiation in air. Science.

[CR123] Wheeler MC, Hendon HH (2004). An all-season real-time multivariate MJO index: development of an index for monitoring and prediction. Mon Weather Rev.

[CR124] WHO (2002). Global solar UV index: a practical guide: a joint recommendation of World Health Organization, World Meteorological Organization, United Nations Environment Programme, International Commission on Non-Ionizing Radiation Protection.

[CR125] Willmott CJ, Robeson SM, Matsuura K (2012). A refined index of model performance. Int J Climatol.

[CR126] Wu K, Wu J, Feng L, Yang B, Liang R, Yang S, Zhao R (2021). An attention-based CNN-LSTM-BiLSTM model for short-term electric load forecasting in integrated energy system. Int Trans Electr Energy Syst.

[CR127] Yadav AK, Chandel SS (2014). Solar radiation prediction using artificial neural network techniques: a review. Renew Sustain Energy Rev.

[CR128] Yan H, Sun L, Wang Y, Huang W, Qiu S, Yang C (2011). A record of the Southern Oscillation Index for the past 2,000 years from precipitation proxies. Nat Geosci.

[CR129] Zhang W, Qu Z, Zhang K, Mao W, Ma Y, Fan X (2017). A combined model based on CEEMDAN and modified flower pollination algorithm for wind speed forecasting. Energy Convers Manag.

[CR130] Zhang J, Zhang X, Niu J, Hu BX, Soltanian MR, Qiu H, Yang L (2019). Prediction of groundwater level in seashore reclaimed land using wavelet and artificial neural network-based hybrid model. J Hydrol.

